# Iron Chelation Therapy in Iron Overload Disorders: A Systematic Review with Narrative Synthesis of Bioenergetic Dysfunction, Oxidative Stress, and Biomarker-Guided Management

**DOI:** 10.3390/ijms27198657

**Published:** 2026-09-28

**Authors:** Khawlah Abdulrahman Salem Alburayh, Ashraf Mahmoud Emara, Rehab Mohamed Elgharabawy

**Affiliations:** 1 Department of Pharmacology and Toxicology, College of Pharmacy, Qassim University, Buraydah 51452, Al Qassim, Saudi Arabia; 2 Department of Pharmacology and Toxicology, Faculty of Pharmacy, Tanta University, Tanta 31511, Egypt; rehab.elgharabawy@pharm.tanta.edu.eg

**Keywords:** iron overload, mitochondria, iron chelation therapy, labile plasma iron, oxidative stress, ferroptosis, β-thalassemia, myelodysplastic syndromes, narrative synthesis, bioenergetics

## Abstract

Iron overload associated with transfusion-dependent disorders promotes oxidative stress, mitochondrial bioenergetic dysfunction, and ferroptotic cell death. Although iron chelation therapy remains the cornerstone of management, the mechanistic links between iron burden, subcellular injury, and emerging biomarkers have not been comprehensively integrated within a clinically relevant framework. This systematic review aimed to synthesize clinical and preclinical evidence on the effects of iron chelation therapy on systemic iron burden, mitochondrial bioenergetics, oxidative stress, ferroptosis, and biomarker-guided management. A systematic literature search was conducted in PubMed/MEDLINE, Embase, Scopus, and Web of Science for studies published from January 2000 to June 2026, following PRISMA 2020 guidelines. The database search yielded 465 records. After removing 120 duplicates, 345 unique records were screened by title and abstract, of which 210 did not satisfy the predefined eligibility criteria. The remaining 135 articles underwent full-text assessment; 45 were excluded because of inappropriate study design (n = 20), lack of relevant outcomes (n = 15), or unavailable full texts (n = 10). Of the 90 remaining articles, 29 studies (7 randomized controlled trials, 6 observational studies, 3 systematic reviews or guidelines, 6 experimental animal studies and 7 in vitro or cellular mechanistic studies) were included for qualitative narrative synthesis. An additional 61 review articles, guidelines, and epidemiological reports were retained for background context. Methodological quality was assessed using the Cochrane Risk of Bias 2, Newcastle–Ottawa Scale, and SYRCLE tools. Clinical evidence indicated that deferoxamine, deferiprone, and deferasirox consistently reduce systemic iron burden, reflected by improvements in serum ferritin, liver iron concentration, and cardiac T2* magnetic resonance imaging. Circulating iron-related biomarkers, including non-transferrin-bound iron and labile plasma iron, provided additional information on redox-active iron exposure. Preclinical evidence further suggested that iron chelation may attenuate mitochondrial dysfunction, oxidative stress, lipid peroxidation, and ferroptotic signaling. However, mechanistic biomarkers of mitochondrial bioenergetics, oxidative injury, and ferroptosis remain insufficiently validated in prospective clinical populations. Considerable heterogeneity in study populations, interventions, biomarker methodologies, and clinical and mechanistic outcomes limited direct quantitative comparison and supported a narrative synthesis approach. Current evidence supports iron chelation as an effective strategy for reducing systemic iron burden, while emerging bioenergetic, redox, and ferroptosis-related biomarkers provide promising mechanistic insights into iron-mediated cellular injury. Nevertheless, their clinical utility remains uncertain because of limited prospective validation, methodological heterogeneity, and insufficient standardization. Future translational studies should integrate conventional iron indices with validated mitochondrial and redox biomarkers and determine whether biomarker-guided chelation strategies can improve clinically meaningful outcomes beyond conventional iron-burden monitoring.

## 1. Introduction

### 1.1. Iron Homeostasis and Iron Overload

Iron is an essential micronutrient supporting oxygen transport, mitochondrial respiration, DNA synthesis, and enzymatic catalysis [1,2]. Systemic iron balance is tightly regulated through intestinal absorption, macrophage recycling, ferritin storage, and the hepcidin–ferroportin axis [3,4] (Figure 1). Disruption of these mechanisms—whether genetic or transfusion-related—overwhelms transferrin-binding capacity, producing non-transferrin-bound iron (NTBI) and redox-active labile plasma iron (LPI). These species drive Fenton-catalyzed oxidative stress, mitochondrial dysfunction, lipid peroxidation, and progressive tissue injury [5,6].

Disruption of these regulatory mechanisms or chronic transfusion therapy can overwhelm the iron-binding capacity of transferrin, resulting in the accumulation of non-transferrin-bound iron (NTBI) and other redox-active iron species. These labile iron pools promote reactive oxygen species generation through Fenton chemistry, leading to oxidative stress, mitochondrial dysfunction, lipid peroxidation, and progressive tissue injury, particularly in the heart, liver, and endocrine organs [7,8].

### 1.2. Physiological Functions of Iron

Iron is indispensable for maintaining cellular metabolism and tissue function through its involvement in oxygen transport, mitochondrial respiration, DNA synthesis, and numerous enzymatic reactions.

#### 1.2.1. Oxygen Transport and Storage

The best-characterized function of iron is its incorporation into the heme group of hemoglobin, where ferrous iron reversibly binds oxygen, allowing efficient oxygen transport from the lungs to peripheral tissues. In skeletal and cardiac muscle, myoglobin serves as an intracellular oxygen reservoir, facilitating oxygen diffusion during periods of increased metabolic demand [1].

#### 1.2.2. Mitochondrial Energy Metabolism

Mitochondria are among the most iron-dependent organelles. Iron-containing heme groups and iron-sulfur (Fe–S) clusters are integral components of electron transport chain complexes, where they mediate electron transfer required for oxidative phosphorylation and ATP generation. Disruption of mitochondrial iron homeostasis impairs electron transport, increases reactive oxygen species (ROS) production, and compromises cellular bioenergetics [9].

The assembly of Fe–S clusters is a highly regulated mitochondrial process involving specialized biosynthetic machinery. These cofactors are required not only for respiratory complexes but also for enzymes involved in intermediary metabolism, DNA maintenance, and cellular signaling. Defects in Fe–S cluster biogenesis result in mitochondrial dysfunction, oxidative stress, and progressive tissue injury [9].

Mitochondrial iron homeostasis is therefore essential for maintaining efficient oxidative phosphorylation and cellular energy production. Under physiological conditions, iron is incorporated into heme groups and iron–sulfur clusters that support electron transfer throughout the respiratory chain. Disruption of this balance leads to impaired ATP generation, electron leakage, and excessive mitochondrial reactive oxygen species (mtROS) production. Persistent mitochondrial oxidative stress further damages mitochondrial proteins, lipids, and mitochondrial DNA, establishing a vicious cycle that progressively amplifies mitochondrial dysfunction and compromises cellular viability [9,10].

#### 1.2.3. DNA Synthesis and Enzymatic Functions

Iron is essential for DNA synthesis through its role as a cofactor for ribonucleotide reductase, the rate-limiting enzyme responsible for deoxyribonucleotide production. Furthermore, numerous enzymes (including cytochrome P450 enzymes, catalase, and peroxidases) depend on iron for catalytic activity, supporting antioxidant defense, xenobiotic metabolism, and multiple metabolic pathways [11].

Collectively, these diverse physiological functions explain why iron homeostasis must be tightly regulated. Even modest disturbances in intracellular iron balance can impair mitochondrial function, compromise redox homeostasis, and initiate mechanisms of cellular injury that contribute to iron overload disorders.

### 1.3. Regulation of Iron Homeostasis

Systemic iron homeostasis is controlled through an integrated regulatory network that coordinates iron absorption, storage, recycling, and cellular utilization. Among these regulatory mechanisms, the hepcidin–ferroportin axis functions as the central determinant of systemic iron balance, while transferrin and ferritin regulate iron distribution and intracellular storage (Figure 2) [1,12].

#### 1.3.1. Hepcidin

Hepcidin is a peptide hormone produced primarily by hepatocytes and is recognized as the master regulator of systemic iron homeostasis. It controls plasma iron concentrations by binding to ferroportin and inducing its internalization and degradation, thereby reducing iron export from enterocytes, macrophages, and hepatocytes. Hepcidin expression increases in response to iron loading and inflammation, whereas hypoxia and increased erythropoietic activity suppress its production to enhance iron availability for erythropoiesis [1,13].

Hepcidin expression is dysregulated in myelodysplastic syndromes, in which ineffective erythropoiesis suppresses hepcidin through erythroferrone-mediated inhibition of BMP–SMAD signaling. A recent systematic review of observational studies confirmed that hepcidin levels in MDS are strongly associated with transfusion burden, serum ferritin, and SF3B1 mutation status [14].

#### 1.3.2. Ferroportin

Ferroportin is the only known cellular iron exporter and is expressed predominantly on enterocytes, macrophages, hepatocytes, and placental cells. Its activity determines the amount of iron released into the circulation and is tightly regulated by hepcidin. Disruption of this interaction results in pathological iron overload or iron-restricted erythropoiesis, highlighting the importance of the hepcidin–ferroportin axis in maintaining systemic iron balance [4].

#### 1.3.3. Transferrin

Transferrin safely transports ferric iron within the circulation and prevents the accumulation of redox-active free iron. Cellular iron uptake occurs through transferrin receptor-mediated endocytosis, while intracellular iron availability regulates transferrin receptor expression via the iron regulatory protein/iron-responsive element (IRP/IRE) system. This coordinated regulation maintains adequate cellular iron supply while limiting excessive iron accumulation [4].

#### 1.3.4. Ferritin

Ferritin functions as the principal intracellular iron storage protein and plays a central role in protecting cells from iron-mediated oxidative damage. During iron deficiency, ferritin undergoes selective lysosomal degradation through ferritinophagy, releasing stored iron for metabolic utilization. In clinical practice, serum ferritin serves as a widely used biomarker of body iron stores, although its interpretation should consider its role as an acute-phase reactant during inflammation [4,15,16,17].

Collectively, these regulatory mechanisms maintain iron concentrations within a narrow physiological range. However, chronic transfusion therapy bypasses intestinal regulatory pathways, producing progressive iron accumulation despite intact homeostatic signaling. This transition from physiological regulation to pathological iron overload underlies the development of transfusion-related organ injury discussed in the following section.

In addition to maintaining physiological iron balance, this regulatory network protects tissues from oxidative injury by minimizing the availability of catalytically active iron. Failure of these regulatory mechanisms results in transferrin saturation, expansion of the labile iron pool, and accumulation of NTBI, thereby facilitating oxidative stress and cellular damage. Consequently, disruption of iron homeostasis represents a key initiating event in the pathogenesis of transfusion-related iron overload [4,7].

**Figure 2:**
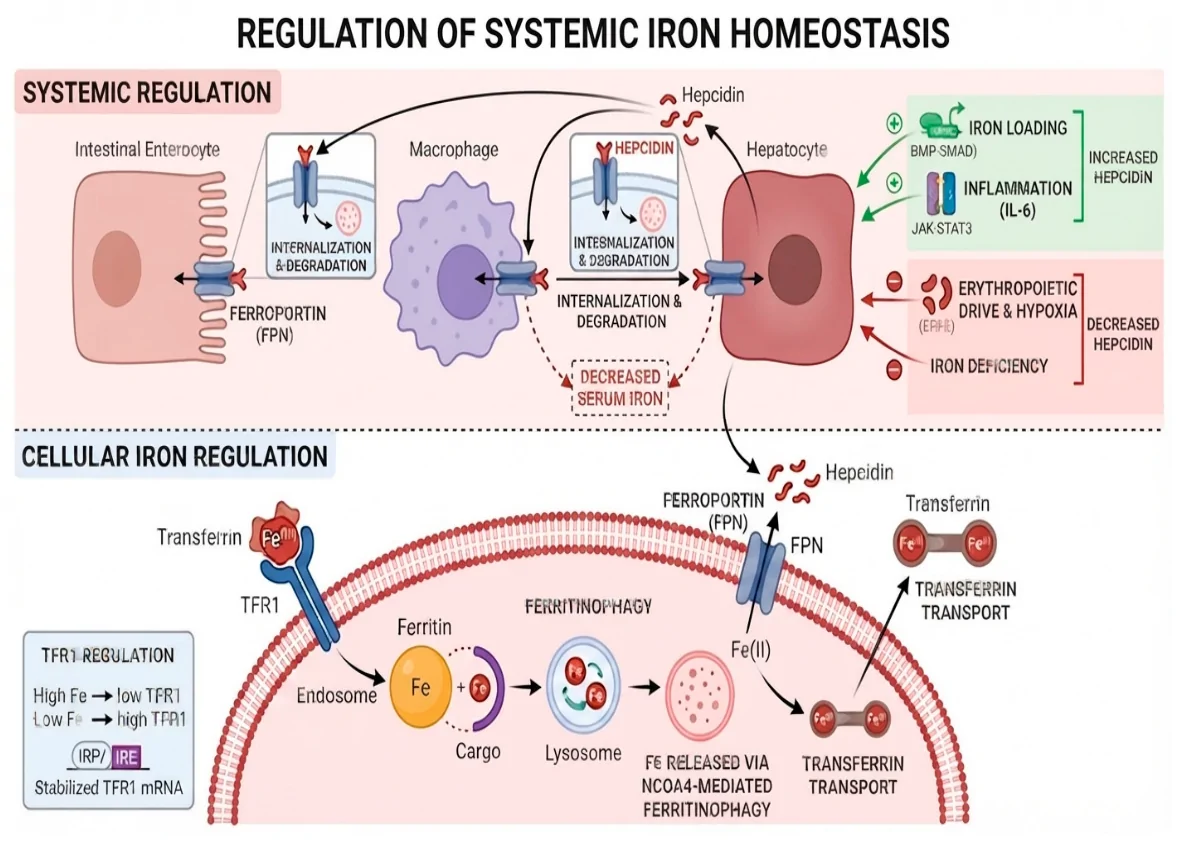
**Regulation of systemic and cellular iron homeostasis.** Hepcidin-mediated regulation of ferroportin coordinates intestinal iron absorption, macrophage iron recycling, and hepatic iron release. Cellular iron balance is maintained through transferrin-mediated uptake, ferritin storage, IRP/IRE regulation of TFR1, and NCOA4-mediated ferritinophagy. Created by the author based on Ganz (2013), Camaschella et al. (2020), Braymer & Lill (2017), Guo et al. (2024) and Sun et al. (2022) [4,5,11,16,17].

### 1.4. Transfusion-Induced Iron Overload: Kinetics and Pathophysiology

Transfusion-dependent disorders, particularly β-thalassemia major and lower-risk myelodysplastic syndromes (MDS), are characterized by progressive iron accumulation resulting from repeated red blood cell transfusions rather than excessive intestinal iron absorption. Each unit of packed red blood cells contains approximately 200–250 mg of elemental iron, and because the human body lacks a regulated mechanism for iron excretion, chronic transfusion therapy inevitably leads to progressive iron overload [18].

Initially, excess iron is stored safely within ferritin; however, continued transfusions eventually exceed the storage capacity of ferritin and transferrin. Once transferrin becomes highly saturated, NTBI appears in the circulation. NTBI is readily taken up by parenchymal cells through poorly regulated pathways, promoting iron accumulation in the liver, heart, pancreas, and endocrine glands independently of normal physiological control [4,18].

The labile plasma iron (LPI) fraction of NTBI is particularly harmful because of its high redox activity. Following cellular uptake, LPI expands the intracellular labile iron pool (LIP), which catalyzes Fenton reactions that generate hydroxyl radicals, initiating oxidative stress, lipid peroxidation, protein oxidation, mitochondrial dysfunction, and ultimately ferroptotic cell death. This labile intracellular iron catalyzes Fenton reactions that generate hydroxyl radicals, initiating oxidative stress, lipid peroxidation, protein oxidation, mitochondrial dysfunction, and ultimately ferroptotic cell death. These molecular events constitute the principal mechanisms underlying iron-induced tissue injury and provide the biological rationale for iron chelation therapy [7,11,19,20].

It is essential to distinguish between three related but distinct iron compartments that are frequently conflated in the literature [20,21,22]:

- **Non-Transferrin-Bound Iron (NTBI):** Iron in the circulation that is not bound to transferrin. NTBI appears when transferrin saturation exceeds approximately 70–80%. It represents a heterogeneous pool comprising both redox-active and relatively inert iron species. NTBI is typically measured by ultrafiltration or high-performance liquid chromatography (HPLC) methods.
- **Labile plasma iron (LPI):** A subset of NTBI that is redox-active and readily available to participate in Fenton chemistry. LPI is the most toxic fraction of circulating iron and represents the primary target of chelation therapy. LPI is measured by the ferrozine assay or fluorescent probes such as calcein-AM.
- **Labile Iron Pool (LIP):** The intracellular pool of loosely bound, redox-active iron that is not sequestered within ferritin or incorporated into iron-containing proteins. Expansion of the LIP drives intracellular oxidative injury. LIP is assessed by calcein-AM fluorescence quenching or electron paramagnetic resonance (EPR) spectroscopy.

These compartments are analytically and biologically distinct. Their conflation in reporting can lead to misinterpretation of chelation efficacy. Throughout this review, we use each term in its specific context: NTBI for circulating unbound iron, LPI for the redox-active plasma fraction, and LIP for the intracellular labile pool.

#### Hepcidin Suppression and Dysregulated Iron Homeostasis

Although iron overload normally stimulates hepcidin production to restrict further iron absorption, this protective response is impaired in transfusion-dependent anemias characterized by ineffective erythropoiesis. Increased erythropoietic activity promotes erythroferrone (ERFE) production, which suppresses hepatic hepcidin synthesis by inhibiting the BMP–SMAD signaling pathway. Consequently, ferroportin activity remains elevated, promoting continued dietary iron absorption and macrophage iron release despite existing systemic iron overload [5].

This pathological suppression of hepcidin creates a self-perpetuating cycle in which transfusional iron loading is compounded by excessive intestinal iron absorption. As a result, iron accumulation progresses even more rapidly, accelerating the development of tissue iron deposition and oxidative injury [4,5]. A recent systematic review of observational studies in MDS confirmed that hepcidin levels are strongly associated with transfusion burden, serum ferritin, and SF3B1 mutation status, underscoring the clinical relevance of this dysregulation [14].

### 1.5. Clinical Impact and Organ Distribution of Transfusion-Related Iron

The clinical manifestations of transfusional iron overload are determined by both the magnitude and distribution of tissue iron deposition. Although the liver serves as the primary storage organ, persistent iron accumulation eventually affects multiple organs, producing progressive structural and functional damage [19].

Hepatic iron overload promotes chronic inflammation, fibrosis, cirrhosis, and increases the long-term risk of hepatocellular carcinoma. In contrast, cardiac iron deposition represents the most life-threatening complication because myocardial iron directly impairs mitochondrial oxidative phosphorylation, ATP production, and cardiac contractility. Clinically, cardiac magnetic resonance imaging (MRI T2*) has become the reference standard for evaluating myocardial iron loading, with lower T2* values strongly associated with increased risks of arrhythmias and heart failure [19,23].

Iron deposition also affects several endocrine organs. Pancreatic iron accumulation contributes to β-cell dysfunction and diabetes mellitus, while pituitary and thyroid involvement may result in hypogonadism, growth hormone deficiency, and hypothyroidism. These complications are particularly common in inadequately chelated patients receiving long-term transfusion therapy [19].

Importantly, many complications of transfusion-related iron overload are preventable or partially reversible when iron accumulation is identified early and treated appropriately. Clinical studies have demonstrated that effective iron chelation therapy reduces tissue iron burden, improves cardiac MRI parameters, restores cardiac function, and decreases iron-related morbidity, emphasizing the importance of early diagnosis and continuous monitoring [24,25,26].

Beyond reducing total body iron burden, early intervention is essential for preserving mitochondrial integrity before irreversible cellular injury occurs. Several studies have demonstrated that timely initiation of chelation therapy not only lowers tissue iron concentrations but also limits oxidative stress, improves mitochondrial bioenergetics, and delays the progression of organ dysfunction. These observations support the concept that mitochondrial impairment is a dynamic and potentially reversible process when iron overload is recognized and treated at an early stage [4,26].

### 1.6. Pathophysiological Mechanisms of Iron Toxicity

The molecular mechanisms underlying iron-mediated cellular injury follow a well-defined sequential cascade. When transferrin saturation exceeds approximately 70–80%, NTBI appears in the circulation [8,27,28]. Within cells, the labile iron pool (LIP)-highly redox-active and poorly sequestered—catalyzes Fenton reactions in which ferrous iron (Fe^2+^) reacts with hydrogen peroxide (H_2_O_2_) to generate hydroxyl radicals (•OH), among the most reactive oxygen species in biological systems [7,12]:

Fe^2+^ + H_2_O_2_ → Fe^3+^ + •OH + OH^−^

Because hydroxyl radicals possess extremely high chemical reactivity and a very short half-life, they damage cellular components immediately after formation [7]. Lipids, proteins, nucleic acids, and mitochondrial membranes are particularly susceptible. Unlike enzymatically generated ROS, hydroxyl radicals cannot be detoxified directly and therefore cause localized but severe molecular damage [7,12].

Lipid peroxidation is one of the earliest consequences. Polyunsaturated fatty acids within cellular and mitochondrial membranes are attacked by hydroxyl radicals, initiating a chain reaction that generates lipid radicals and lipid peroxyl radicals [12,19]. This process disrupts membrane architecture, increases permeability, alters ion transport, and impairs membrane-associated protein function [12,29]. Secondary products include malondialdehyde (MDA) and 4-hydroxynonenal (4-HNE), stable biomarkers of oxidative membrane damage [9,29].

Mitochondrial dysfunction develops as excess mitochondrial iron disrupts iron–sulfur (Fe–S) cluster biogenesis, impairs electron transport chain (ETC) complexes, and promotes continuous mtROS generation [9,11]. Electron leakage from damaged ETC complexes creates a self-amplifying cycle: oxidative damage opens the mitochondrial permeability transition pore (mPTP), collapses mitochondrial membrane potential (ΔΨm), reduces ATP synthesis, and further increases ROS production [9,30].

Redox imbalance occurs as chronic ROS production overwhelms endogenous antioxidant defenses, including reduced glutathione (GSH), superoxide dismutase (SOD), catalase (CAT), and glutathione peroxidase (GPX) [12,19]. Depletion of GSH reduces glutathione peroxidase 4 (GPX4) activity, the principal enzyme responsible for detoxifying membrane lipid hydroperoxides [19,31].

Ferroptosis represents the terminal convergence of these pathways: iron-dependent accumulation of lipid peroxides exceeds cellular antioxidant capacity, resulting in regulated cell death characterized by mitochondrial shrinkage, increased membrane density, and loss of cristae architecture [19,31,32]. This cascade—Fenton-catalyzed ROS generation → lipid peroxidation → mitochondrial dysfunction → ATP depletion → antioxidant exhaustion → ferroptosis—constitutes the central pathophysiological mechanism of transfusion-induced iron overload [31,33]. This sequential cascade is illustrated in Figure 3.

Iron chelation therapy interrupts this cascade by binding redox-active iron, reducing hydroxyl radical formation, attenuating lipid peroxidation, preserving mitochondrial membrane potential, and limiting ferroptotic signaling [7,19,35,36].

## 2. Results

### 2.1. Study Selection

The database search yielded 465 records. After removal of 120 duplicate records, 345 unique records remained for title and abstract screening, of which 210 were excluded because they did not satisfy the predefined eligibility criteria. The remaining 135 articles underwent full-text assessment. Of these, 45 were excluded because of inappropriate study design (n = 20), lack of relevant outcomes (n = 15), or unavailable full texts (n = 10). The remaining 90 articles were subsequently classified according to their role in the review. Of these, 29 studies fulfilled the predefined criteria for inclusion in the qualitative narrative synthesis, whereas 61 review articles, guidelines, and epidemiological reports were retained separately to provide contextual and background evidence and were not included in the qualitative synthesis. Thus, the 90 articles remaining after full-text assessment comprised 29 studies included in the qualitative synthesis and 61 contextual sources. The complete study-selection process is summarized in the PRISMA 2020 flow diagram (Figure 4).

### 2.2. Characteristics of Included Studies

The 29 included studies comprised 7 randomized controlled trials (RCTs), 6 observational or retrospective studies, 3 systematic reviews or guidelines retained for contextual evidence, 6 experimental animal studies, and 7 mechanistic in vitro or cellular studies. The 13 preclinical investigations reported in the Abstract correspond to the 6 experimental animal studies and 7 mechanistic in vitro or cellular studies enumerated here; the 3 systematic reviews or guidelines included in the narrative synthesis met the predefined eligibility criteria by providing pooled clinical evidence or consensus recommendations directly relevant to chelation outcomes, whereas the remaining 61 review articles, guidelines, and epidemiological reports were retained exclusively for background context and were not incorporated into the qualitative synthesis. Clinical investigations were conducted across North America, Europe, Asia, the Middle East, and Australia, and enrolled patients with transfusion-dependent β-thalassemia, sickle cell disease, hereditary hemochromatosis, myelodysplastic syndromes, and aplastic anemia. Preclinical studies utilized rodent models of iron overload cardiomyopathy, hepatocyte cultures, neuronal cell lines, and erythroid progenitors. The evaluated iron chelation therapies included deferoxamine (DFO), deferiprone (DFP), deferasirox (DFX), and combination regimens. Clinical studies predominantly reported iron burden outcomes (serum ferritin, liver iron concentration, cardiac MRI T2*, NTBI, and LPI), whereas preclinical studies focused on oxidative stress biomarkers, mitochondrial bioenergetics, and ferroptosis-related parameters. The distribution and characteristics of included studies are summarized in Table 1, and study-level details are provided in Table 2.

### 2.3. Clinical Evidence: Iron Burden and Organ-Specific Outcomes

The 14 clinical studies consistently demonstrated that iron chelation therapy reduced systemic and organ-specific iron burden across diverse transfusion-dependent disorders, although the magnitude of response varied according to baseline iron overload, treatment duration, and chelation regimen. The principal therapeutic interventions evaluated were deferoxamine (DFO), deferiprone (DFP), and deferasirox (DFX), administered either as monotherapy or in combination. Clinical efficacy was assessed using established biomarkers of iron overload, including serum ferritin, liver iron concentration (LIC), cardiac magnetic resonance imaging T2* (MRI T2*), non-transferrin-bound iron (NTBI), and labile plasma iron (LPI) [24,25,42,45,62].

#### 2.3.1. Systemic Iron Burden

Serum ferritin was the most frequently reported indicator of treatment response. Across randomized controlled trials and prospective cohort studies, sustained iron chelation therapy was associated with progressive reductions in serum ferritin concentrations, particularly among patients with transfusion-dependent β-thalassemia receiving DFX or DFP-containing regimens. Similar improvements were reported in patients with myelodysplastic syndromes and other chronic transfusion-dependent anemias, although the magnitude of reduction depended on baseline iron burden and cumulative transfusion exposure [24,39,63,64].

Liver iron concentration (LIC), measured by magnetic resonance imaging or liver biopsy, showed corresponding reductions following prolonged chelation therapy. Improvements in LIC were consistently observed after 12–24 months of treatment and generally paralleled changes in serum ferritin, supporting the effectiveness of iron chelation in reducing hepatic iron accumulation [39,63,64].

#### 2.3.2. Cardiac Iron Overload

Eight clinical investigations evaluated myocardial iron using cardiac MRI T2*, the current non-invasive reference standard for cardiac iron assessment. Patients receiving continuous chelation therapy generally demonstrated stabilization or improvement in myocardial T2* values, indicating reduced cardiac iron deposition. Studies evaluating deferiprone-containing regimens reported greater improvements in cardiac iron removal than deferoxamine alone, while combination therapy produced the most pronounced improvements in patients with severe myocardial siderosis [43,65,66,67].

Several studies also documented improvements in left ventricular function following reductions in myocardial iron, although cardiac functional outcomes were reported less consistently than imaging-based measurements [68,69].

#### 2.3.3. Circulating Toxic Iron Species

Five studies assessed circulating toxic iron species, including NTBI and LPI, which represent catalytically active forms of plasma iron capable of promoting oxidative injury. Iron chelation therapy consistently reduced both NTBI and LPI concentrations, with decreases occurring rapidly after treatment initiation and preceding reductions in total body iron stores. Combination chelation regimens generally achieved greater suppression of circulating toxic iron than monotherapy, particularly in heavily transfused patients [70,71,72,73,74].

#### 2.3.4. Clinical Outcomes

Clinical outcomes were reported in nine studies and included changes in transfusion requirements, cardiac complications, hepatic function, endocrine abnormalities, and treatment-related adverse events. Long-term chelation therapy was associated with lower rates of iron-related organ dysfunction and stabilization of cardiac disease in patients achieving sustained reductions in iron burden. Most studies reported acceptable safety profiles for the three approved chelators, although gastrointestinal adverse effects, renal function changes, neutropenia, and agranulocytosis were observed in a minority of patients, consistent with the established safety profiles of these agents [75,76,77,78,79,80].

#### 2.3.5. Overall Clinical Evidence

Despite heterogeneity in study populations, disease characteristics, treatment protocols, and follow-up duration, the included clinical studies consistently demonstrated that iron chelation therapy effectively reduced systemic and tissue iron burden. Improvements were observed across multiple complementary biomarkers, including serum ferritin, LIC, cardiac MRI T2*, NTBI, and LPI, providing convergent evidence of therapeutic efficacy. The strongest evidence originated from randomized controlled trials and multi-center prospective studies, whereas smaller observational investigations generally supported these findings across a broader range of transfusion-dependent disorders [18,28,37].

### 2.4. Preclinical Evidence: Subcellular Mechanisms

The 13 preclinical investigations (6 animal and 7 cellular studies) examined mitochondrial bioenergetics, oxidative stress, and ferroptotic signaling under iron overload and chelation conditions. Experimental models reported that iron chelation was associated with increased ATP production, stabilization of mitochondrial membrane potential (ΔΨm), and enhanced activity of respiratory chain complexes. Chelation-mediated reduction in redox-active iron decreased mitochondrial ROS generation and lipid peroxidation products, including malondialdehyde, in selected cell-based assays. Several experimental studies further indicated that iron chelation modulated ferroptotic signaling through partial restoration of glutathione peroxidase 4 (GPX4) activity and reduction in lipid hydroperoxide accumulation. These mechanistic findings were observed under controlled experimental conditions utilizing acute or subacute iron-loading protocols.

### 2.5. Risk of Bias and Certainty of Evidence

Risk-of-bias assessments are presented in Supplementary Table S1 and Supplementary Figure S1. Among the 7 RCTs, 2 were rated as low overall risk of bias [25,80], while 5 raised some concerns related to open-label designs, missing outcome data, or absence of pre-registered protocols. Of the 6 observational studies, 2 achieved good quality ratings on the Newcastle–Ottawa Scale (7–8 stars), whereas the remaining 4 were rated as moderate quality (4–6 stars). All 13 preclinical studies were rated as unclear risk of bias on the SYRCLE tool due to inadequate reporting of randomization, allocation concealment, and blinding. The 3 systematic reviews and guidelines were not formally assessed with RoB 2, NOS, or SYRCLE.

Using the GRADE framework, certainty of evidence was rated as moderate for serum ferritin reduction across chelators, low for cardiac T2* improvement, and very low for mechanistic outcomes (mitochondrial function, oxidative stress, and ferroptosis) because the latter evidence derived exclusively from preclinical studies with unclear risk of bias and lack of direct clinical validation. The GRADE summary is presented in Supplementary Table S2.

## 3. Discussion

### 3.1. Principal Findings

This systematic review synthesized 29 studies to evaluate the effects of iron chelation therapy on systemic iron burden and subcellular mechanisms of iron-mediated injury. The evidence supports a hierarchical model of chelation-mediated protection. At the clinical level, deferoxamine, deferiprone, and deferasirox consistently reduce systemic iron burden, as reflected by improvements in serum ferritin, liver iron concentration, and cardiac MRI T2* values [24,25,36,39,81]. These macro-level benefits are well established across randomized controlled trials and large cohorts. At the mechanistic level, preclinical investigations provide biological plausibility that chelation attenuates mitochondrial dysfunction, reduces oxidative stress, and limits ferroptotic signaling [9,19,31]. However, these subcellular mechanisms have not been conclusively validated as independent drivers of clinical outcome in human patients, and the extent to which they represent therapeutic pathways versus downstream consequences of iron removal remains uncertain.

### 3.2. Clinical Efficacy and Macro-Level Outcomes

The clinical findings summarized in Section 2.3 support a hierarchical benefit across chelators. Rather than repeating these macro-level outcomes here, we focus on two interpretive considerations. First, while serum ferritin and LIC provide validated metrics of total body iron, they do not capture the redox-active iron fraction (NTBI/LPI) directly responsible for oxidative injury [20,28]. Second, cardiac T2* improvement with deferiprone and early-initiated deferasirox suggests that myocardial iron clearance is both agent-dependent and time-sensitive, yet head-to-head RCTs comparing all three chelators specifically for cardiac outcomes remain limited [18,81].

Several studies also investigated circulating forms of labile iron, particularly NTBI and LPI. Clinical evidence generally demonstrated reductions in circulating NTBI and LPI following effective iron chelation, suggesting that these biomarkers reflect the biologically active iron fraction responsible for oxidative injury more accurately than serum ferritin alone [20,82]. Nevertheless, their routine clinical use remains limited by the lack of standardized analytical methods and restricted assay availability across clinical laboratories. It is essential to distinguish between these compartments: NTBI reflects circulating unbound iron, LPI identifies the redox-active fraction responsible for plasma oxidative injury, and the intracellular labile iron pool represents the metabolically active iron that directly mediates mitochondrial dysfunction and ferroptosis [8,20]. These pools are analytically and biologically distinct, and their conflation in reporting can lead to misinterpretation of chelation efficacy. A detailed comparison of their analytical and biological characteristics is provided in Table 3. The principal clinical and circulating biomarkers used to monitor systemic iron burden and their expected response to iron chelation therapy are summarized in the systemic iron burden section of Table 4.

### 3.3. Mechanistic Insights from Preclinical Evidence

In contrast to the robust clinical evidence for macro-level iron removal, insights into subcellular mechanisms derive predominantly from experimental investigations. Rather than reiterating the pathophysiological cascade detailed in Section 1.6 and Figure 5, the following synthesis focuses on the specific molecular loci at which iron chelation interrupts iron-mediated cellular injury.

Preclinical studies utilizing cellular and animal models of iron overload reported directional improvements in mitochondrial bioenergetic parameters following chelation exposure, including increased ATP production, stabilization of mitochondrial membrane potential (ΔΨm), and enhanced activity of respiratory chain complexes [9,48,49]. These findings suggest that reduction in intracellular labile iron may alleviate mitochondrial oxidative stress and partially restore oxidative phosphorylation capacity. Experimental models further indicated that chelation attenuates lipid peroxidation and modulates ferroptotic signaling, evidenced by decreased malondialdehyde formation and partial restoration of glutathione peroxidase 4 (GPX4) activity in selected cell-based assays [19,29].

As illustrated in Figure 5, the three approved chelators—deferoxamine (DFO), deferiprone (DFP), and deferasirox (DFX)—bind redox-active iron at distinct compartments, including circulating labile plasma iron (LPI), intracellular labile iron pools, and mitochondrial iron overload, thereby attenuating downstream ROS generation, lipid peroxidation, and ferroptotic signaling [19,35]. The major biomarkers used to assess redox homeostasis, lipid peroxidation, and antioxidant defense in iron overload disorders, and their expected response to chelation therapy, are summarized in the oxidative stress and antioxidant defense sections of Table 4.

However, it is critical to emphasize that these observations were made under controlled experimental conditions—often involving acute or subacute iron-loading protocols that do not replicate the chronic, decade-long tissue iron accumulation experienced by human patients. Consequently, the extent to which these mechanistic benefits translate into clinically meaningful improvements in organ function remains uncertain.

### 3.4. The Translational Gap: From Bench to Bedside

Despite major advances in iron chelation therapy, an important translational gap persists. Current clinical assessment relies primarily on conventional macro-level biomarkers such as serum ferritin, liver iron concentration, and cardiac MRI [8,25]. Although these parameters provide valuable information regarding systemic and organ-specific iron burden, they do not directly evaluate real-time micro-cellular recovery, mitochondrial function, or intracellular oxidative stress, which represent the actual mechanisms underlying iron-mediated cytotoxicity [9].

Growing experimental evidence indicates that mitochondrial dysfunction develops early during iron overload, contributing directly to impaired cellular bioenergetics and progressive organ injury. Nevertheless, relatively few clinical studies have comprehensively evaluated whether macro-level improvements in iron burden following chelation therapy are accompanied by corresponding subcellular restoration of mitochondrial function and redox homeostasis. The relationship between systemic iron removal and the true reversibility of intracellular bioenergetic failure remains incompletely understood [9].

Similarly, while oxidative stress is recognized as a central driver of iron-induced cellular injury, available clinical evidence regarding the capacity of individual chelators to restore redox homeostasis remains inconsistent. Variability in study design, patient populations, treatment regimens, and biomarker selection has limited direct comparison among previous investigations [12]. Among currently available biomarkers, malondialdehyde, reduced glutathione, the GSH/GSSG ratio, and antioxidant enzyme activities are the most frequently evaluated indicators of therapeutic response. Nevertheless, considerable variability in analytical methods and sampling protocols has limited standardization across studies [12,29].

Ferroptosis represents another area where the translational gap is pronounced. Although substantial experimental evidence supports ferroptosis as a central mechanism of iron-mediated cellular injury in vitro and in animal models, its precise contribution to organ dysfunction in human transfusion-dependent iron overload remains incompletely defined [19,31]. Most mechanistic studies have been conducted in cultured cells or rodent models exposed to acute iron loading—conditions that may not fully replicate the chronic, heterogeneous, and multi-organ nature of transfusion-dependent iron overload in patients. Furthermore, currently available clinical studies rely largely on indirect biomarkers of lipid peroxidation and oxidative stress rather than validated ferroptosis-specific biomarkers suitable for human application [57,60]. Consequently, it remains uncertain whether suppression of ferroptosis directly translates into improved clinical outcomes or merely reflects reduced oxidative injury as a downstream consequence of iron removal.

### 3.5. Biomarker-Guided Management: Current Status and Future Directions

The integration of subcellular biomarkers into routine therapeutic monitoring remains preliminary. While MRI-derived liver iron concentration and cardiac T2* provide accurate, validated assessments of tissue-level iron accumulation, their correlation with real-time mitochondrial function, intracellular oxidative stress, or ferroptotic activity has not been established in prospective clinical cohorts [9,25].

Biomarkers such as ATP production, mitochondrial membrane potential, mitochondrial ROS, and mitochondrial DNA copy number were evaluated almost exclusively in preclinical investigations or small translational pilot studies with limited sample sizes and short follow-up durations [9,12]. A comprehensive integrated framework of systemic iron burden, oxidative stress, antioxidant defense, mitochondrial bioenergetics, biogenesis, and ferroptosis-related biomarkers is presented in Table 4. These biomarkers show conceptual promise for deepening mechanistic understanding and may eventually complement conventional iron monitoring, but their clinical utility for guiding treatment decisions or predicting organ-specific outcomes requires rigorous validation through standardized, harmonized assays in large, multi-center prospective studies.

Future clinical investigations should employ harmonized biomarker panels that integrate conventional oxidative stress markers with mitochondrial biomarkers to provide a more comprehensive assessment of iron-induced cellular injury and recovery following chelation therapy. Shifting toward such an integrated approach is critical to moving past passive macro-iron monitoring and establishing an active, biomarker-guided framework for organelle protection and precision dosing.

### 3.6. Therapeutic Considerations and Emerging Strategies

Current international guidelines recommend initiating iron chelation before irreversible organ injury develops. Early intervention has been shown to reduce tissue iron accumulation, improve long-term survival, and substantially decrease the incidence of cardiac complications [8,18]. Successful management requires an individualized approach integrating the degree of iron accumulation, transfusion requirements, organ involvement, patient age, treatment adherence, and drug tolerability (Figure 6).

**Figure 6:**
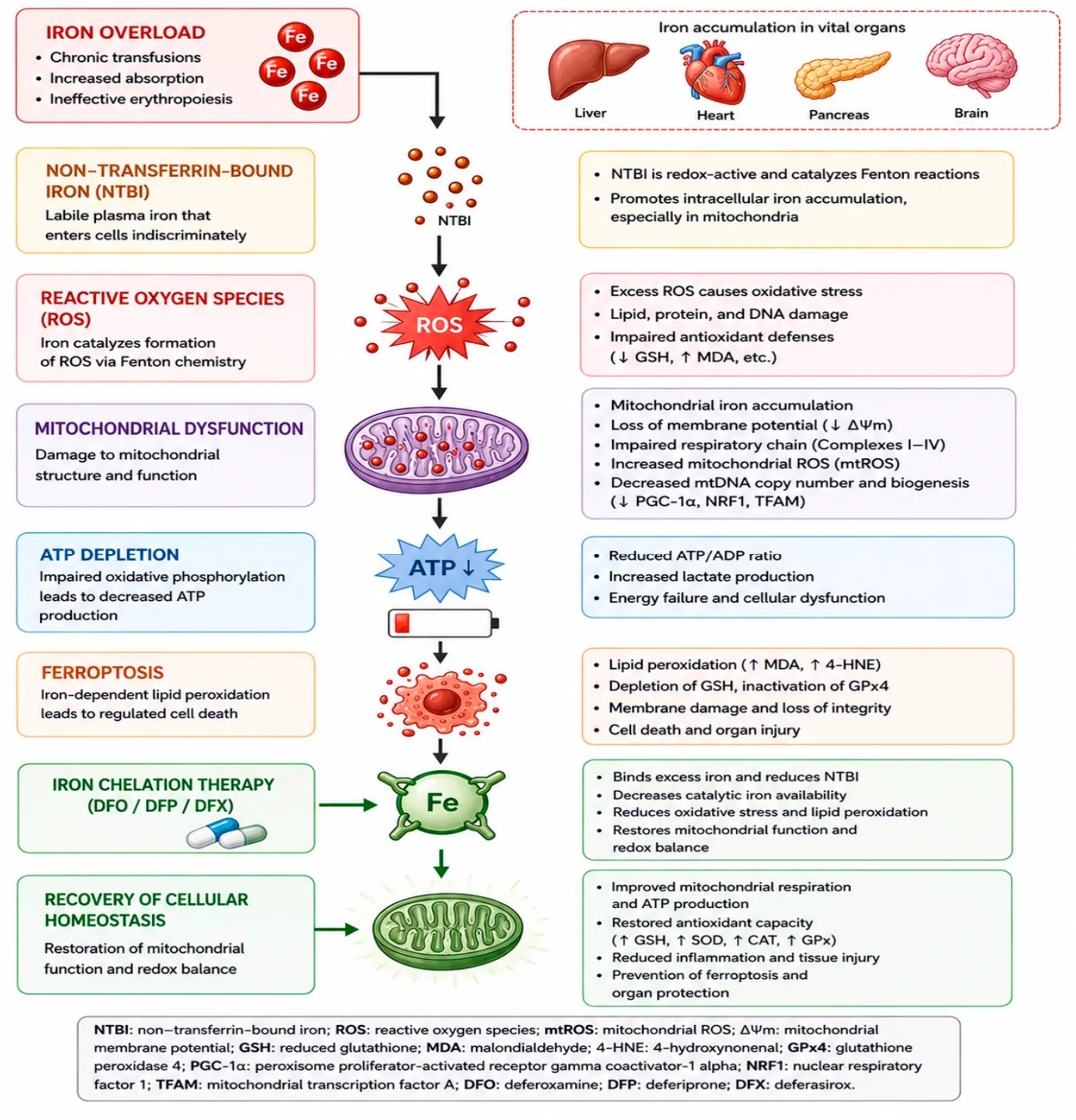
Mechanisms of action, pharmacological characteristics, and mitochondrial protective effects of the three clinically approved iron chelators: deferoxamine (DFO), deferiprone (DFP), and deferasirox (DFX). The pharmacological properties, dosing, tissue distribution, and adverse effect profiles of each chelator are detailed in Section 4.6 and Table 5. All three chelators reduce systemic iron burden by binding redox-active iron; deferiprone demonstrates superior myocardial penetration, while deferasirox provides sustained plasma exposure via once-daily oral dosing. Created by the author based on Cappellini et al. (2021), Taher et al. (2017), and Angelucci et al. (2020) [8,18,24].

**Table 5:** Comparative Characteristics, Pharmacological Properties, and Mitochondrial Protective Effects of Approved Iron Chelators.

Characteristic	Deferoxamine (DFO)	Deferiprone (DFP)	Deferasirox (DFX)
**Route**	Subcutaneous/Intravenous infusion [8,10]	Oral [8,10]	Oral [8,10]
Chelation Stoichiometry	Hexadentate (binding)	Bidentate (ligand: binding)	Tridentate (ligand: binding)
Typical dosing	20–60 mg/kg/day [8,10]	75–100 mg/kg/day [8,10]	20–40 mg/kg/day [8,10]
Plasma half-life	20–30 min [35]	3–4 h [35]	8–16 h [35]
Primary iron removal	Hepatic (Taher & Saliba, 2017; [18])	Cardiac [10,25]	Hepatic and cardiac [10,25]
Clinical indications	Severe transfusional iron overload [8]	Cardiac siderosis or combination therapy [8,25]	First-line long-term iron chelation [8,10]
Major adverse effects	Ocular toxicity, ototoxicity, infusion-site reactions [35]	Agranulocytosis, neutropenia [35]	Renal impairment, hepatic dysfunction, gastrointestinal adverse effects [37]
Monitoring	Audiology, ophthalmology, serum ferritin [8]	Weekly complete blood count and serum ferritin [8]	Serum creatinine, liver enzymes, serum ferritin [8]
Representative clinical trials	EPIC [38]	Randomized controlled trial/interventional clinical trial [25]	EPIC [38]; TELESTO [24]; ECLIPSE [37]
Combination therapy	Yes [35,36]	Yes [35,36]	Yes [36,45]
Approximate annual drug cost	Low–Moderate [10,40]	Moderate [10,40]	High [10,40]
Cost-effectiveness considerations	Lower acquisition cost but higher administration burden [40]	Cost-effective in patients with cardiac iron overload an as combination therapy [25,45]	Higher acquisition cost may be offset by greater adherence and reduced long-term complications [10,37,40]

The three approved chelators differ in pharmacological characteristics that influence clinical selection. Deferoxamine remains highly effective for reducing hepatic iron stores but requires prolonged subcutaneous infusion, which limits adherence [8,35]. Deferiprone offers superior myocardial penetration and is particularly valuable for patients with established cardiac iron overload, though it requires monitoring for agranulocytosis [25,35]. Deferasirox provides sustained plasma exposure via convenient once-daily oral dosing, improving adherence, and has demonstrated efficacy in both hepatic and cardiac iron removal [25,37,38]. Combination therapy has shown particular benefit in patients with severe cardiac siderosis or inadequate response to monotherapy, where more aggressive iron removal is required [36,45].

From a pharmacoeconomic perspective, effective chelation therapy substantially reduces the incidence of cardiac disease, liver failure, endocrine dysfunction, and other costly complications associated with chronic iron overload. Prevention of these complications decreases hospitalization rates and improves quality of life, supporting the concept that optimization of chelation therapy should be regarded as an investment in prevention rather than solely as a pharmacological expense [40]. However, long-term adherence remains a major challenge, and simplifying treatment regimens represents a major objective of modern management [67].

Although currently available chelators have markedly improved survival, important clinical challenges remain. Variability in patient adherence, differences in tissue-specific iron clearance, treatment-related adverse effects, and incomplete reversal of established organ damage continue to limit therapeutic outcomes. Emerging strategies include optimization of combination chelation, nanotechnology-based delivery systems to improve intracellular targeting [52], and therapeutic approaches combining iron chelation with mitochondria-targeted antioxidants or ferroptosis inhibitors. Because oxidative stress and mitochondrial dysfunction contribute substantially to iron-mediated tissue injury, simultaneous targeting of these interconnected pathways may provide greater protection than iron removal alone [9,12].

Novel drug delivery systems may improve chelator bioavailability and tissue distribution. Nanochelator formulations, for example, have demonstrated enhanced biodistribution and targeting in experimental iron overload models [52]. Additionally, intranasal deferoxamine has shown increased brain exposure and neuroprotection in ischemic stroke models, illustrating how alternative delivery routes might expand organ-specific protection [50]. Targeting chelatable iron has also provided neuroprotection in Parkinson’s disease models, further supporting the concept that tissue-specific iron removal can yield clinical benefits beyond systemic depletion [51].

Looking further ahead, the hepcidin–ferroportin axis represents a primary target for novel therapeutic designs. In transfusion-dependent hemoglobinopathies, ineffective erythropoiesis suppresses endogenous hepcidin, resulting in hyperabsorption of dietary iron. Synthetic hepcidin agonists and direct ferroportin inhibitors offer alternative approaches to restrict iron entry into the plasma pool [5,53]. Mitochondria-targeted antioxidants such as MitoQ and SkQ1 accumulate selectively within the mitochondrial matrix, scavenging mitochondrial ROS, preserving respiratory chain complexes, and mitigating iron-induced mitochondrial DNA damage [34,87,88]. These strategies are supported by observational evidence that hepcidin levels in MDS correlate with transfusion burden and genetic status, suggesting that hepcidin-directed therapy may be particularly relevant in transfusion-dependent MDS [14]. Complementing this, small-molecule ferroptosis inhibitors—including ferrostatin-1 derivatives and agents targeting the FSP1-CoQ and DHODH pathways—act as lipophilic radical-trapping antioxidants that interrupt lipid peroxyl propagation, potentially protecting cardiac and hepatic parenchyma when deployed alongside standard chelators [58,59,60].

Curative genetic interventions offer the potential to eliminate the fundamental cause of iron loading. Autologous hematopoietic stem cell modification via lentiviral gene addition or CRISPR/Cas9 editing of the *BCL11A* enhancer can reactivate fetal hemoglobin production, enabling transfusion independence and shifting the clinical paradigm from managing active iron loading to gradually depleting established tissue iron [89].

### 3.7. Strengths and Limitations

This review has several notable strengths. The methodology followed PRISMA 2020 guidelines, ensuring a comprehensive and reproducible literature search and study selection process. Evidence was synthesized from multiple major biomedical databases, and the review integrates clinical, biochemical, and mechanistic evidence, offering a perspective that links iron chelation therapy with bioenergetic dysfunction, oxidative stress, and biomarker-guided management.

Several limitations should be acknowledged. The included studies exhibited considerable heterogeneity in study design, patient populations, iron overload etiologies, treatment regimens, follow-up duration, and outcome measures, which precluded quantitative meta-analysis. Differences in the biomarkers used to assess oxidative stress and mitochondrial dysfunction limited direct comparisons across studies. Furthermore, many mechanistic insights were derived from experimental and preclinical investigations; although these findings provide important biological evidence, their direct clinical applicability requires further validation in large prospective studies. Finally, publication bias and language bias cannot be completely excluded because only studies published in English and indexed in the selected databases were included.

### 3.8. Conclusion and Future Perspectives

Current clinical evidence robustly supports the established role of iron chelation therapy with deferoxamine, deferiprone, and deferasirox in reducing systemic iron burden and preventing iron-mediated organ injury in transfusion-dependent patients. These benefits are reflected by improvements in serum ferritin, liver iron concentration, and cardiac MRI T2* values—macro-level parameters that remain the standard of care for monitoring treatment efficacy [24,25,38].

Experimental evidence from cellular and animal models suggests that iron chelation may additionally attenuate mitochondrial dysfunction, reduce oxidative stress, and limit ferroptotic cell death [9,19,31]. These mechanistic findings provide important biological plausibility for the observed clinical benefits. However, these subcellular mechanisms have not been consistently validated in large prospective clinical cohorts. Direct clinical evidence linking chelation-induced improvements in mitochondrial bioenergetics to patient-centered outcomes remains limited, and the preclinical studies supporting these mechanisms utilized acute iron-loading protocols that differ substantially from chronic human disease.

Emerging biomarkers of mitochondrial function and redox homeostasis show conceptual promise and may eventually complement conventional iron monitoring. Nevertheless, their clinical utility requires rigorous prospective validation through standardized, harmonized assays in large, multi-center clinical trials. Similarly, hepcidin-based biomarkers may refine risk stratification in MDS, as elevated hepcidin levels have been associated with transfusion dependence and SF3B1 mutation status in systematic analyses [14]. Future research should prioritize well-designed translational studies that integrate conventional iron indices with validated mitochondrial functional assays, and randomized controlled trials evaluating whether biomarker-guided chelation strategies improve clinically meaningful endpoints beyond current standard care. Until such evidence is available, clinical management of transfusion-dependent iron overload should continue to rely on established macro-level parameters, while mechanistic biomarkers remain valuable research tools awaiting clinical validation.

## 4. Materials and Methods

### 4.1. Study Selection and PRISMA Flow Synthesis

The database search yielded 465 records. After removing 120 duplicates, 345 unique records underwent independent title and abstract screening by two reviewers, of which 210 were excluded for not satisfying the predefined eligibility criteria. The remaining 135 articles were assessed in full text; 45 were excluded because of inappropriate study design (n = 20), lack of relevant outcomes (n = 15), or unavailable full texts (n = 10). This left 90 articles, of which 29 studies fulfilled the inclusion criteria for qualitative narrative synthesis. The remaining 61 articles—comprising review articles, guidelines, and epidemiological reports—were retained separately as background sources to support contextual interpretation and were not included in the qualitative synthesis. The complete study-selection process is summarized in the PRISMA 2020 flow diagram (Figure 4). Disagreements at any stage were resolved through discussion and consensus, with consultation of a third reviewer when necessary.

### 4.2. Database Search Strategy and Information Sources

A comprehensive electronic literature search was conducted across PubMed/MEDLINE, Embase, Scopus, and Web of Science to identify relevant studies published between January 2000 and June 2026. The search strategy combined controlled vocabulary, including Medical Subject Headings (MeSH) and Emtree terms where appropriate, with free-text keywords related to iron overload, iron chelation therapy, mitochondrial dysfunction, oxidative stress, ferroptosis, and associated biomarkers. Boolean operators (AND/OR) were used to combine search terms and maximize retrieval of relevant publications.

The search strategy included three major concept domains: (1) iron overload disorders (e.g., “iron overload,” “transfusion-dependent,” “β-thalassemia,” and “myelodysplastic syndromes”); (2) mechanisms of iron-mediated cellular injury (e.g., “mitochondrial dysfunction,” “bioenergetics,” “oxidative stress,” “ferroptosis,” “reactive oxygen species,” and “mitochondrial ROS”); and (3) iron chelation therapy (e.g., “iron chelation,” “deferoxamine,” “deferiprone,” and “deferasirox”). Detailed search strategies for each database are presented in Table 6.

To ensure comprehensive study identification, the reference lists of eligible articles and relevant review papers were manually screened for additional studies not retrieved through the electronic database search.

### 4.3. Study Selection and Eligibility Criteria

Eligible studies included randomized controlled trials, non-randomized clinical studies, observational studies, experimental animal investigations, and mechanistic cellular studies evaluating iron overload or transfusion-related iron accumulation. Studies were required to report at least one relevant outcome from either of the following categories: (a) Clinical iron burden biomarkers: serum ferritin, liver iron concentration, cardiac MRI T2*, transferrin saturation, NTBI, or LPI; (b) Mechanistic biomarkers of iron-mediated cellular injury: mitochondrial bioenergetics (ATP production, mitochondrial membrane potential, respiratory chain complex activity), oxidative stress markers (reactive oxygen species, lipid peroxidation products, antioxidant enzyme activities), or ferroptosis-related parameters. Studies were not required to report outcomes from both categories simultaneously.

### 4.4. Inclusion and Exclusion Parameters

Studies were included if they were published in English, involved clinically relevant or experimentally validated models of iron overload, and reported at least one outcome related to either: (i) iron burden (e.g., serum ferritin, liver iron concentration, cardiac magnetic resonance imaging [MRI T2*], transferrin saturation, NTBI, LPI); or (ii) iron-mediated cellular injury mechanisms (e.g., mitochondrial bioenergetics [ATP production, mitochondrial membrane potential, respiratory chain complex activity], oxidative stress markers [reactive oxygen species, lipid peroxidation products, antioxidant enzyme activities], or ferroptosis-related parameters).

Studies were not required to report outcomes from both categories simultaneously. It was anticipated that clinical studies (randomized controlled trials and observational studies) would predominantly report iron burden outcomes, whereas experimental animal and cellular studies would predominantly report mechanistic outcomes. This divergence in biomarker reporting across study designs reflects the current state of the evidence base and was expected a priori. Studies reporting only clinical biomarkers or only mechanistic biomarkers were therefore eligible for inclusion, provided they met all other criteria.

The following publications were excluded: editorials, commentaries, conference abstracts without accessible full texts, letters, dissertations, case reports, studies unrelated to iron overload or iron chelation therapy, investigations of iron deficiency or non-iron metal toxicities, studies evaluating unapproved experimental chelators without clinical relevance, and duplicate publications.

### 4.5. Quantitative Filtration and PRISMA Flow Synthesis

Of the 135 full-text articles assessed for eligibility, 45 were excluded because of inappropriate study design (n = 20), lack of relevant outcomes (n = 15), or unavailable full texts (n = 10). Consequently, 90 articles remained after full-text assessment. Of these, 29 studies fulfilled the predefined eligibility criteria and were included in the qualitative narrative synthesis, whereas 61 review articles, guidelines, and epidemiological reports were retained separately as background sources to support contextual interpretation and were not included in the qualitative synthesis. Thus, the 90 articles remaining after full-text assessment comprised 29 studies included in the qualitative synthesis and 61 contextual sources. The complete study-selection process is summarized in the PRISMA 2020 flow diagram (Figure 4).

### 4.6. Data Extraction Protocols and Critical Appraisal

Data extraction was performed independently by two reviewers using a standardized extraction form. Information collected from each study included the first author, publication year, country, study design, participant population or experimental model, underlying iron overload condition, intervention or chelation regimen, biomarkers evaluated, primary clinical or mechanistic outcomes, and principal study conclusions. Discrepancies in data extraction were resolved through discussion until consensus was achieved.

Methodological quality was assessed according to study design. Randomized controlled trials were evaluated using the Cochrane Risk of Bias 2 (RoB 2) tool [90], observational studies were assessed using the Newcastle–Ottawa Scale (NOS) [91], and experimental animal and cellular studies were evaluated using the SYRCLE Risk of Bias tool [92]. The complete risk-of-bias assessments are presented in Supplementary Table S1. The results of the quality assessment were considered during interpretation of the evidence, with greater weight given to findings from studies with lower risk of bias, but were not used as criteria for study exclusion.

### 4.7. Rationale for Narrative Synthesis and Preclusion of Quantitative Meta-Analysis

Quantitative meta-analysis was precluded because the included studies exhibited extreme and multifactorial heterogeneity across four principal dimensions. Any attempt at statistical pooling would have violated the fundamental assumption of a common underlying effect size, rendering pooled estimates statistically unreliable and clinically misleading. The specific sources of heterogeneity are detailed below.

#### 4.7.1. Clinical Heterogeneity

The included primary studies enrolled markedly diverse patient and experimental populations. Clinical studies involved patients with transfusion-dependent β-thalassemia, sickle cell disease, hereditary hemochromatosis, myelodysplastic syndromes (MDS), and aplastic anemia. These populations differed in: (i) underlying disease pathophysiology; (ii) transfusion burden and duration; (iii) age distribution (pediatric versus adult cohorts); (iv) baseline iron status; (v) comorbidity profiles (cardiac dysfunction, hepatic impairment, endocrine disorders); and (vi) prior chelation exposure. Preclinical studies utilized heterogeneous experimental models, including rodent iron overload cardiomyopathy, hepatocyte cultures, neuronal cell lines, and erythroid progenitors, with varying iron loading protocols (acute versus chronic, parenteral versus dietary iron administration). The clinical and biological diversity across these populations precludes the assumption that a single, common treatment effect exists.

#### 4.7.2. Intervention Heterogeneity

Chelator regimens varied substantially across studies in: (i) agent selection (deferoxamine [DFO], deferiprone [DFP], deferasirox [DFX], or combination therapy); (ii) dosing schedules (continuous subcutaneous infusion, thrice-daily oral, once-daily oral); (iii) routes of administration (subcutaneous, intravenous, oral); (iv) treatment durations (ranging from single-dose cellular experiments to multi-year clinical trials); and (v) concomitant therapies (transfusion protocols, antioxidant supplementation, other disease-modifying agents). These variations in intervention intensity, pharmacokinetic exposure, and tissue distribution introduce substantial effect-modifier heterogeneity that cannot be adequately addressed through subgroup meta-analysis given the limited number of studies within each stratum.

#### 4.7.3. Outcome Heterogeneity

The biomarkers and clinical endpoints assessed across studies differed markedly in: (i) analytical methodology (e.g., malondialdehyde [MDA] measured by thiobarbituric acid reactive substances [TBARS] versus high-performance liquid chromatography [HPLC]; ATP measured by luciferase assay versus HPLC; mitochondrial membrane potential [ΔΨm] assessed by JC-1 versus tetramethylrhodamine methyl ester [TMRM] fluorescence); (ii) tissue or fluid source (serum, plasma, erythrocytes, urine, liver biopsy, cardiac tissue, isolated mitochondria, cultured cells); (iii) reporting units (absolute concentrations, ratios, percentages, arbitrary fluorescence units); and (iv) timing of measurement relative to intervention onset. This methodological heterogeneity precludes meaningful standardization of effect sizes across studies.

#### 4.7.4. Design Heterogeneity

The evidence base comprised a mixture of: randomized controlled trials (n = 7), observational, cohort, and retrospective studies (n = 6), systematic reviews and guidelines (n = 3), animal experiments (n = 6), and in vitro cellular investigations (n = 7). The intrinsic differences in internal validity, risk of bias, and generalizability across these design categories further undermine the assumption of a common effect size underlying meta-analytic models.

#### 4.7.5. Statistical Implications

Given the extreme clinical, methodological, and statistical heterogeneity described above—spanning diverse populations, interventions, outcomes, and designs—quantitative pooling would be inappropriate. Forest plots would likely reveal non-overlapping confidence intervals, further confirming that meta-analytic aggregation is not justified. Both random-effects (DerSimonian–Laird or restricted maximum likelihood) and fixed-effects inverse-variance models would violate their foundational assumptions. A random-effects model would inappropriately generalize beyond the observed studies, whereas a fixed-effects model would assume a single true effect that is clearly biologically implausible given the clinical and methodological diversity. Meta-regression to explore heterogeneity sources would be underpowered given the small number of studies within each covariate stratum.

#### 4.7.6. Consistency with Guideline Frameworks

The decision to employ narrative synthesis is fully consistent with Cochrane guidance [93] and the PRISMA 2020 statement [94], both of which explicitly recognize narrative synthesis as a valid and appropriate methodology when statistical pooling is precluded by heterogeneity. The revised title, abstract, and methods sections of this manuscript now transparently communicate this methodological choice to readers.

### 4.8. Use of Artificial Intelligence

During the preparation of this manuscript, the authors used Gemini (Google LLC, Mountain View, CA, USA, version 3.5) as a writing assistant to help organize the structure, improve readability, and refine the English language. This tool was used strictly for editorial and stylistic support; all scientific content, data interpretation, and conclusions were developed, verified, and approved by the authors, who retain full responsibility for the intellectual integrity and accuracy of the final manuscript.

## 5. Conclusions

In conclusion, current clinical evidence robustly supports the established role of iron chelation therapy with deferoxamine, deferiprone, and deferasirox in reducing systemic iron burden and preventing iron-mediated organ injury in transfusion-dependent patients. These benefits are reflected by improvements in serum ferritin, liver iron concentration, and cardiac MRI T2* values— macro-level parameters that remain the standard of care for monitoring treatment efficacy and are validated across large randomized controlled trials and prospective cohorts.

Experimental evidence from cellular and animal models suggests that iron chelation may additionally attenuate mitochondrial dysfunction, reduce oxidative stress, and limit ferroptotic cell death. These mechanistic findings provide important biological plausibility for the observed clinical benefits of chelation therapy. However, it must be emphasized that these subcellular mechanisms have not been consistently validated in large prospective clinical cohorts. Direct clinical evidence linking chelation-induced improvements in mitochondrial bioenergetics—specifically ATP production, mitochondrial membrane potential (ΔΨm), and mitochondrial ROS—to patient-centered outcomes such as survival, cardiac function, or quality of life remains limited. The preclinical studies supporting these mechanisms utilized acute or subacute iron-loading protocols that differ substantially from the chronic, decade-long tissue iron accumulation experienced by transfusion-dependent patients. Consequently, the extent to which mitochondrial protection, ferroptosis modulation, and redox homeostasis restoration represent independent therapeutic pathways versus downstream consequences of iron removal in human patients remains uncertain.

Emerging biomarkers of mitochondrial function and redox homeostasis show conceptual promise for deepening mechanistic understanding and may eventually complement conventional iron monitoring in clinical practice. Nevertheless, their clinical utility for guiding treatment decisions, predicting organ-specific outcomes, or enabling precision dosing requires rigorous prospective validation through standardized, harmonized assays in large, multi-center clinical trials. Future research should prioritize well-designed translational studies that integrate conventional iron indices with validated mitochondrial functional assays, and randomized controlled trials evaluating whether biomarker-guided chelation strategies improve clinically meaningful endpoints beyond current standard care. Until such evidence is available, clinical management of transfusion-dependent iron overload should continue to rely on established macro-level parameters—serum ferritin, liver iron concentration, and cardiac MRI T2*—while mechanistic biomarkers remain valuable research tools awaiting clinical validation.

## Figures and Tables

**Figure 1:**
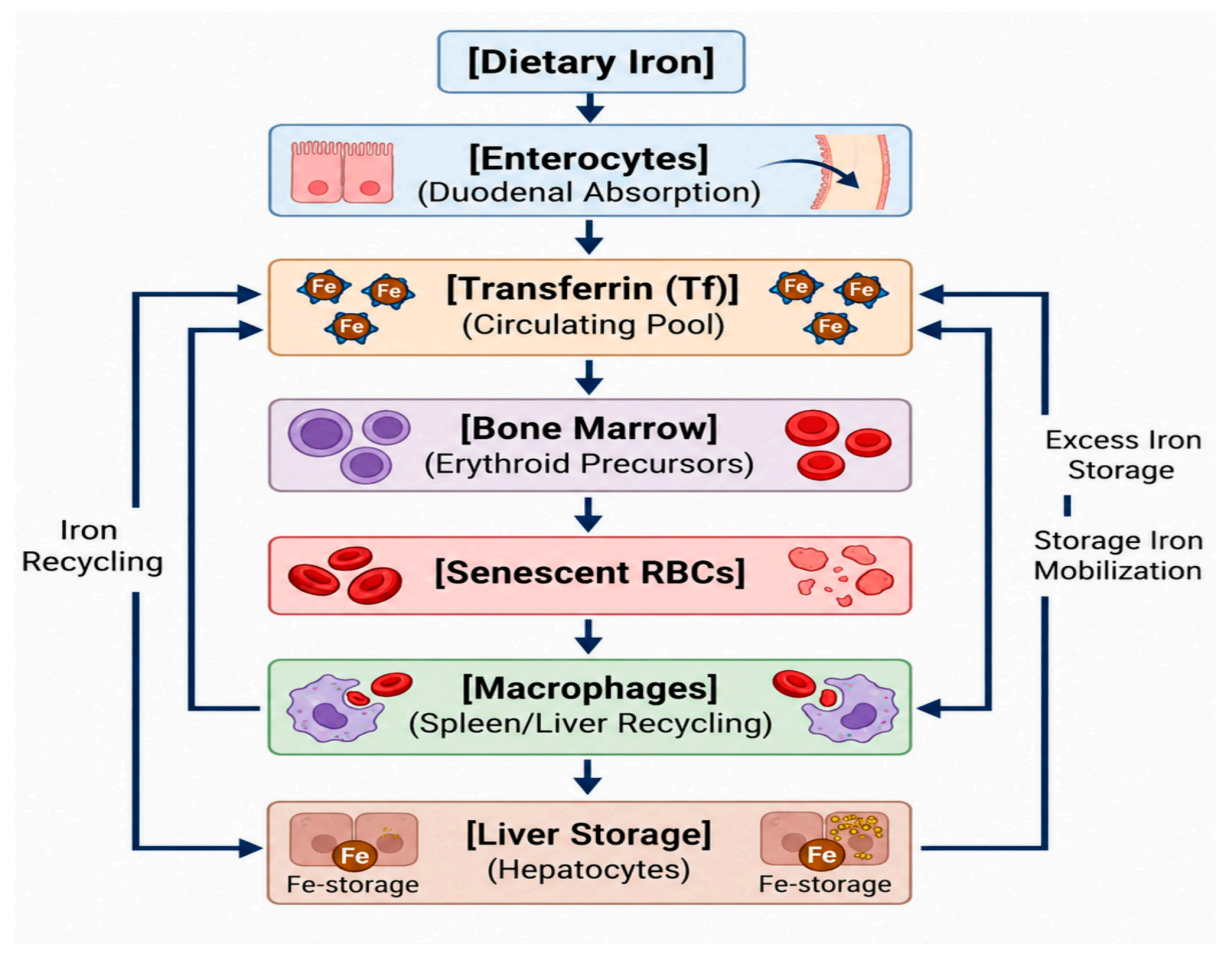
Schematic representation of systemic iron homeostasis and the internal iron cycle. Dietary iron absorbed via duodenal enterocytes enters the systemic circulation bound to transferrin. This circulating pool is primarily delivered to the bone marrow for erythropoiesis. Senescent erythrocytes are subsequently cleared by tissue macrophages, which recycle iron back into the circulation. Excess iron is stored within hepatocytes in the liver, which acts as the primary systemic reservoir and regulates homeostasis through the hormone hepcidin.

**Figure 3:**
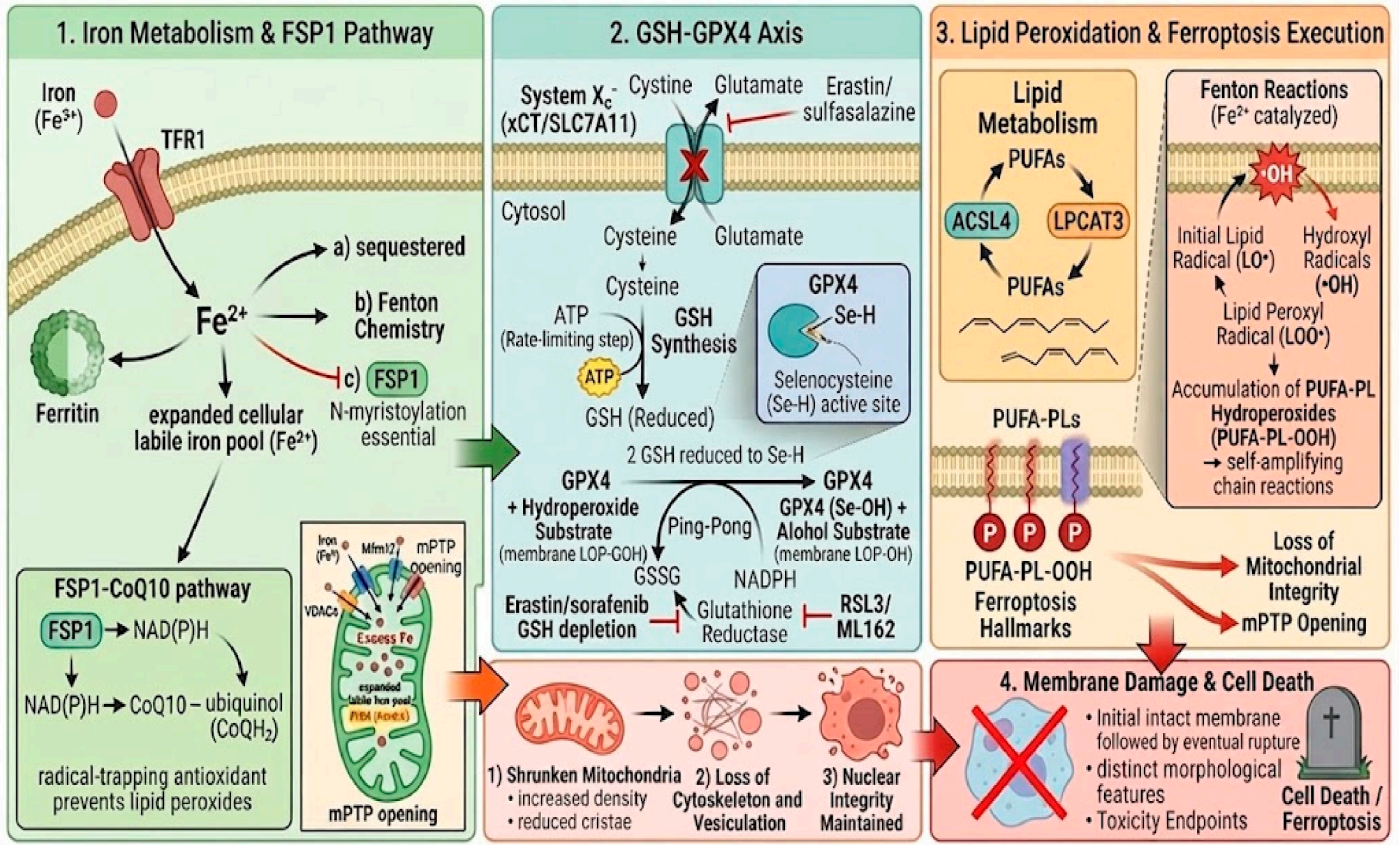
Sequential pathophysiology of transfusion-induced iron overload. Chronic red blood cell transfusions saturate transferrin binding capacity, leading to NTBI and labile plasma iron (LPI) accumulation. These redox-active iron species catalyze Fenton reactions, generating hydroxyl radicals that instigate lipid peroxidation, mitochondrial membrane damage, electron transport chain inhibition (notably Complex IV), ATP depletion, antioxidant exhaustion, and ultimately ferroptosis. The fundamental mechanisms underlying this cascade are described in Section 1.6. Created by the author based on Stockwell et al. (2017), Dixon et al. (2012), Jiang et al. (2020), and Chen et al. (2021) [31,32,33,34].

**Figure 4:**
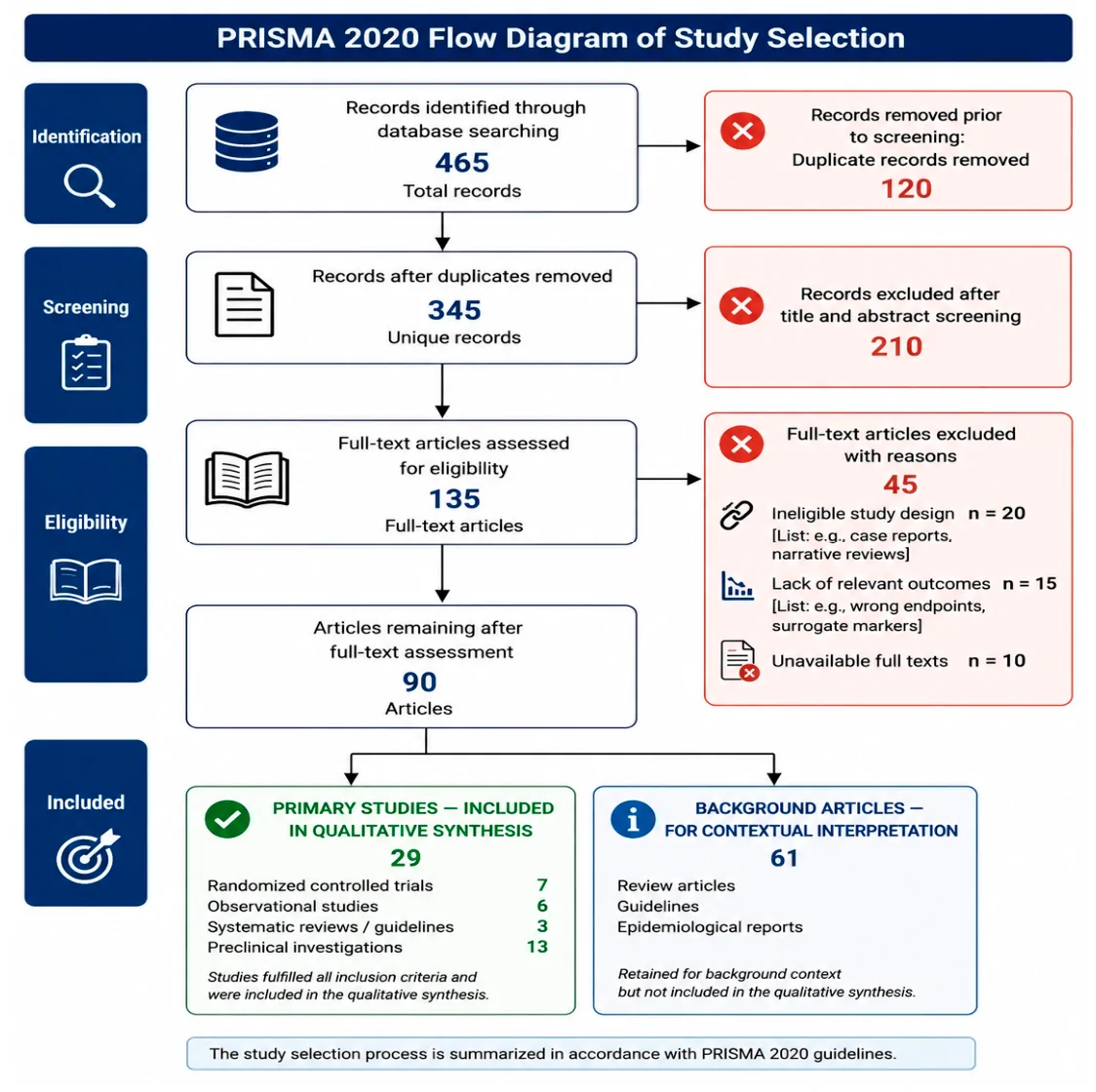
PRISMA flow diagram detailing the systematic literature selection process.

**Figure 5:**
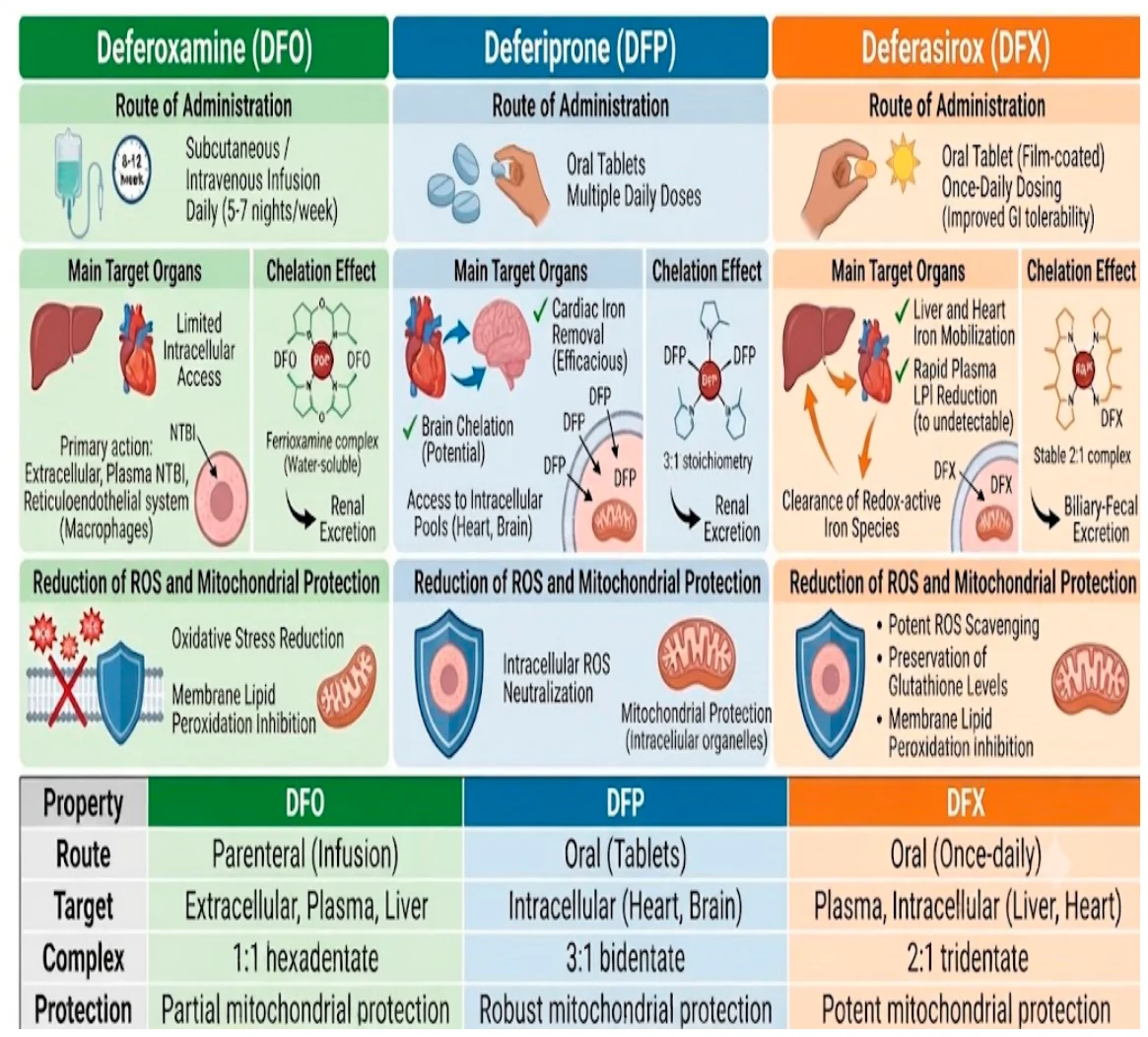
Therapeutic intervention points of iron chelation within the iron overload pathophysiological cascade. Deferoxamine (DFO), deferiprone (DFP), and deferasirox (DFX) bind redox-active iron at specific loci—circulating labile plasma iron (LPI), intracellular labile iron pools, and mitochondrial iron compartments—thereby interrupting Fenton chemistry, attenuating lipid peroxidation, preserving mitochondrial membrane potential (ΔΨm), and limiting ferroptotic cell death. The upstream pathophysiological cascade (transferrin saturation → NTBI → LPI → ROS → mitochondrial dysfunction → ferroptosis) is detailed in Figure 3 and Section 1.6. Created by the author based on Stockwell et al. (2017), Dixon et al. (2012), Fang et al. (2019), and Kontoghiorghes & Kontoghiorghe (2020) [19,31,32,35].

**Table 1:** Overview of Included Study Characteristics (n = 29).

Characteristic	Description
**Number of primary studies**	29
Publication period	2000–2026
Study designs	7 randomized controlled trials; 6 observational, cohort, or retrospective studies; 3 systematic reviews or guidelines; 6 experimental animal studies; 7 in vitro or cellular mechanistic studies
Geographical distribution	North America, Europe, Asia, the Middle East, and Australia
Primary disease populations	Transfusion-dependent β-thalassemia, sickle cell disease, hereditary hemochromatosis, myelodysplastic syndromes, aplastic anemia, and secondary transfusional iron overload
Iron chelation therapies investigated	Deferoxamine (DFO), deferiprone (DFP), deferasirox (DFX), and combination regimens
Primary clinical outcomes	Serum ferritin, liver iron concentration (LIC), cardiac T2* MRI, transferrin saturation, NTBI, LPI
Mechanistic outcomes	Oxidative stress biomarkers, mitochondrial bioenergetics (ATP, respiratory complexes), ferroptosis parameters, inflammatory mediators, and redox homeostasis

**Table 2:** Study-Level Evidence Summary of the 29 Primary Studies.

No.	Study (Author, Year)	Design	Population/Model	Intervention/Exposure	Key Outcomes	Principal Findings
1	Słomka et al., 2024 [14]	Systematic review	MDS patients	N/A (hepcidin assessment)	Hepcidin, ferritin, SF3B1, transfusion burden	Hepcidin suppressed in MDS; levels correlate with transfusion burden and mutation status
2	Angelucci et al., 2020 [24]	RCT	Low- to intermediate-1-risk MDS (n = 224)	Deferasirox vs. placebo	Serum ferritin, LIC, cardiac T2*, OS	Reduced ferritin; improved event-free survival vs. placebo
3	Pennell et al., 2010 [25]	RCT	β-Thalassemia major	Deferasirox (up to 3 years)	Cardiac T2*	Sustained deferasirox improved myocardial T2* and prevented cardiac siderosis
4	Premawardhena et al., 2024 [36]	RCT (open-label)	β-Thalassemia with very high iron overload	Triple therapy (DFO + DFP + DFX)	Serum ferritin, LIC, cardiac T2*	Rapid iron reduction in severe overload
5	Pennell et al., 2012 [37]	RCT	β-Thalassemia major	Deferiprone vs. deferoxamine	Cardiac T2*, myocardial iron	Deferiprone demonstrated superior myocardial iron removal
6	Taher et al., 2017 [38]	RCT	Thalassemia or lower-risk MDS	Deferasirox film-coated tablet	Tolerability, serum ferritin, safety	Well tolerated with maintained efficacy
7	Cappellini et al., 2010 [39]	RCT	Transfusion-dependent anemias (n = 1744)	Deferasirox tailored to iron intake	Serum ferritin, LIC, safety	Effective tailored chelation across diverse populations
8	Pongamnuaykrit et al., 2025 [40]	Prospective cohort	Transfusion-dependent thalassemia (second-line)	Deferasirox monotherapy	Efficacy, safety, ferritin, LIC	Effective as second-line therapy after prior chelator failure
9	Radhwi et al., 2025 [41]	Retrospective cost analysis	Transfusion-dependent β-thalassemia (Saudi Arabia)	Various chelation regimens	Direct medical costs	Quantified annual illness burden and adherence-related cost drivers
10	Anderson et al., 2001 [42]	Observational cohort	Transfusion-dependent thalassemia	Myocardial iron monitoring (cardiac MRI T2*)	Myocardial T2*, cardiac function	Quantified myocardial iron overload; correlation with decreased LVEF
11	Pennell et al., 2006 [43]	Clinical trial/cohort	Transfusion-dependent anemias	Chelation guided by myocardial T2* MRI	Myocardial iron clearance, cardiac morbidity	Targeted cardiac iron reduction lowers heart failure risk
12	Taher et al., 2010 [44]	Observational study	Beta-thalassemia intermedia	Assessment of systemic iron overload and morbidities	Thromboembolic events, pulmonary hypertension, leg ulcers	Iron-related complications occur independently of transfusion dependence
13	Cappellini et al., 2006 [45]	RCT	Transfusion-dependent thalassemia	Deferasirox vs. deferoxamine	LIC, serum ferritin	Comparable efficacy and safety of oral chelation
14	Salem et al., 2023 [46]	Systematic review	β-Thalassemia major	DFP + DFX combination	Efficacy, safety, cardiac T2*	Combination oral therapy effective for cardiac and hepatic iron reduction
15	Memish & Saeedi, 2011 [47]	Retrospective program data	Saudi national premarital screening cohort	N/A	Disease prevalence, screening uptake	Established epidemiological burden of hemoglobinopathies in Saudi Arabia
16	Manoguerra et al., 2005 [48]	Consensus guideline	Iron ingestion patients	Deferoxamine (out-of-hospital)	Management protocols, toxicity thresholds	Evidence-based consensus for out-of-hospital iron poisoning management
17	Fefelova et al., 2023 [49]	Animal (mouse)	Cardiac iron overload model	MCU deficiency	Cardiac function, ferroptosis markers	MCU abrogation protected against iron-overload cardiomyopathy
18	Sripetchwandee et al., 2014 [50]	Animal (rat)	Cardiac iron overload model	DFO/MCU blockade	Mitochondrial Ca^2+^, ROS, membrane potential	MCU blockade prevented cardiac mitochondrial dysfunction
19	Hanson et al., 2009 [51]	Animal (rat)	Ischemic stroke model	Intranasal deferoxamine	Brain DFO exposure, infarct volume	Intranasal delivery increased brain DFO exposure and conferred neuroprotection
20	Devos et al., 2014 [52]	Animal (mouse)	Parkinson’s disease model	Deferoxamine	Chelatable iron, oxidative stress, motor function	Targeting chelatable iron provided neuroprotection
21	Jones et al., 2020 [53]	Animal (rodent)	Iron overload model	DFO-based nanochelators	Iron clearance, biodistribution, toxicity	Nanochelator formulations improved bioavailability and tissue targeting
22	Manolova et al., 2020 [54]	Animal (mouse)	β-Thalassemia model	VIT-2763 (ferroportin inhibitor)	Ineffective erythropoiesis, splenomegaly, iron loading	Oral ferroportin inhibitor ameliorated ineffective erythropoiesis
23	Fang et al., 2023 [55]	In vitro (cardiomyocytes)	Mouse cardiomyocytes	Iron overload/chelation/ferroptosis inhibitors	Ferroptosis, lipid peroxidation, cell death	Ferroptosis is a targetable mechanism of iron-mediated cardiomyocyte injury
24	Battaglia et al., 2020 [56]	In vitro (cancer cell lines)	Various cancer cell lines	Iron modulation, erastin, RSL3	Ferroptosis, mitochondrial morphology, iron	Convergence of mitochondrial iron metabolism and ferroptotic cell death
25	Doll et al., 2017 [57]	In vitro (cancer cell lines)	Various cancer cell lines	ACSL4 modulation/knockout	Lipid composition, ferroptosis sensitivity	ACSL4 dictates ferroptosis sensitivity by shaping lipid composition
26	Kraft et al., 2020 [58]	In vitro (cancer cell lines)	Various cancer cell lines	GCH1 overexpression/knockout	Tetrahydrobiopterin, lipid remodeling, ferroptosis	GCH1/tetrahydrobiopterin counteracts ferroptosis through lipid remodeling
27	Mao et al., 2021 [59]	In vitro (cancer cell lines)	Various cancer cell lines	DHODH inhibition	Ferroptosis, CoQ10, mitochondrial respiration	DHODH-mediated ferroptosis defense is a targetable vulnerability
28	Bersuker et al., 2019 [60]	In vitro (cancer cell lines)	Various cancer cell lines	FSP1 modulation/knockout	CoQ oxidoreductase activity, ferroptosis	FSP1 acts parallel to GPX4 to inhibit ferroptosis via CoQ reduction
29	Xiao et al., 2025 [61]	In vitro (cardiomyocytes)	Cardiomyocytes (iron overload)	Parkin overexpression/ACSL4 ubiquitination	Ferroptosis, PUFA-phospholipid metabolism	Parkin inhibits iron-overload cardiomyocyte ferroptosis by ubiquitinating ACSL4

**Table 3:** Comparison of non-transferrin-bound iron (NTBI), labile plasma iron (LPI), and the intracellular labile iron pool (LIP) in iron overload disorders.

Characteristic	Non-Transferrin-Bound Iron (NTBI)	Labile Plasma Iron (LPI)	Intracellular Labile Iron Pool (LIP)
**Definition**	Circulating iron not bound to transferrin that appears after transferrin saturation is exceeded [27,81,83]	Redox-active fraction of NTBI capable of catalyzing reactive oxygen species generation [83,84]	Metabolically active, chelatable intracellular iron available for cellular metabolism [20,85]
Biological compartment	Plasma [27]	Plasma [84]	Cytoplasm and intracellular organelles, particularly mitochondria [83,86]
Formation	Develops when transferrin becomes saturated during iron overload [64,81]	Represents the redox-active component of NTBI [83,84]	Generated through dysregulated intracellular iron homeostasis and increased cellular iron uptake [86]
Chemical characteristics	Heterogeneous mixture of low-molecular-weight iron complexes [27,81]	Highly reactive catalytic iron capable of participating in Fenton chemistry [84]	Dynamic intracellular iron pool supporting physiological and pathological cellular processes [85]
Primary biological role	Source of tissue iron uptake through non-transferrin pathways [27,83]	Mediates oxidative stress through reactive oxygen species generation [83,84]	Supports normal mitochondrial metabolism but promotes oxidative injury and ferroptosis when expanded [83,86]
Major pathological significance	Promotes iron deposition in the heart, liver, pancreas, and endocrine organs [27,64]	Drives lipid peroxidation and oxidative tissue injury [84]	Promotes mitochondrial dysfunction, impaired ATP production, and ferroptosis [20,83]
Analytical methods	Chelation-based assays, HPLC, or other specialized laboratory techniques [27]	Fluorescence-based assays measuring redox-active iron [84]	Fluorescent probes (e.g., calcein-AM, FerroOrange) used primarily in experimental research [20]
Routine clinical use	Limited to specialized laboratories [81]	Mainly research and selected specialized centers [84]	Not routinely measured clinically [20]
Clinical utility	Assessment of systemic iron toxicity and response to chelation [8,81]	Assessment of circulating redox-active iron and short-term chelation efficacy [84]	Investigation of cellular iron homeostasis and mechanisms of mitochondrial injury [86]
Relationship to iron chelation	Generally decreases following effective iron chelation therapy [8,63]	Rapidly decreases with successful chelation because of reduced catalytic iron [84]	Experimental studies suggest normalization following reduction in intracellular iron burden [86]
Current evidence	Well supported by clinical and translational studies [8,27]	Supported by clinical and translational studies, although assay standardization remains limited [81]	Supported predominantly by experimental animal and cellular investigations [20,83]

**Table 4:** Integrated Biomarker Framework for Iron Overload Assessment and Chelation Response Monitoring.

Category	Biomarker	Biological Significance	Expected Change in Iron Overload	Effect of Effective Chelation	Clinical/Research Utility
Systemic Iron Burden	Serum ferritin [24,25,38]	Intracellular iron storage protein; surrogate for total body iron stores	↑	↓ Reduced	Standard clinical marker for systemic iron burden and chelation response. Limited specificity during inflammation.
	Liver iron concentration (LIC) [25,36]	Direct measure of hepatic iron accumulation by MRI or biopsy	↑	↓ Reduced	Reference standard for hepatic iron loading; guides chelation intensity and duration.
	Cardiac MRI T2* [25,26]	Non-invasive myocardial iron assessment; lower values reflect higher tissue iron burden	↓ T2*	↑ Improved T2*	Clinical reference standard for cardiac iron; predicts arrhythmia and heart failure risk.
	NTBI [10,20]	Circulating iron not bound to transferrin; appears when transferrin saturation exceeds ~70–80%	↑	↓ Reduced	Reflects systemic iron toxicity and chelatable iron pool; more specific than ferritin for redox-active iron.
	LPI [20,84]	Redox-active fraction of NTBI capable of catalyzing Fenton reactions	↑	↓ Reduced	Represents the most toxic circulating iron fraction and the primary pharmacological target of chelation therapy.
Oxidative Stress & Lipid Peroxidation	Total ROS [7,12]	Global indicator of intracellular oxidative stress and redox imbalance	↑	↓ Reduced	Standard marker to evaluate systemic and tissue-specific oxidant burden.
	mtROS [9,12]	Localized mitochondrial superoxide production and respiratory electron leakage	↑	↓ Reduced	Sensitive marker for early-stage mitochondrial damage and dysfunction.
	MDA [12,29]	Stable end-product of polyunsaturated fatty acid degradation via lipid peroxidation	↑	↓ Reduced	Most commonly used plasma and tissue biomarker for lipid peroxidation.
	4-HNE [7,29]	Highly reactive cytotoxic aldehyde generated during lipid peroxidation	↑	↓ Reduced	Stable marker of severe oxidative membrane damage and protein modification.
	Protein carbonyls [9,12]	Irreversible oxidative modifications formed during structural oxidation of proteins	↑	↓ Reduced	Standard benchmark to quantify downstream functional damage to the cellular proteome.
	8-OHdG [9,19]	Major product of oxidative DNA damage from hydroxyl radical attack	↑	↓ Suppressed	Essential index of genomic and mitochondrial DNA integrity under genotoxic conditions.
	Nitrotyrosine [12,19]	Product of protein tyrosine nitration mediated by peroxynitrite	↑	↓ Reduced	Marker of simultaneous oxidative and nitrosative stress driving vascular inflammation.
Antioxidant Defense	GSH [12,19]	Primary endogenous low-molecular-weight intracellular antioxidant	↓	↑ Restored	Critical biomarker of remaining cellular reductive and buffering capacity.
	GSSG [12,19]	Oxidized glutathione dimer accumulated during radical detoxification	↑	↓ Reduced	Evaluates active antioxidant utilization and free-radical scavenging stress.
	GSH/GSSG ratio [12,19]	Master parameter of intracellular and mitochondrial redox state	↓	↑ Restored	Robust metric of oxidative stress and tissue recovery.
	SOD [7,12]	Dismutates superoxide radicals into hydrogen peroxide	↓	↑ Restored	Assesses intermediate enzymatic defense capacity against early superoxide production.
	CAT [7,12]	Converts hydrogen peroxide into water and oxygen	↓	↑ Restored	Evaluates enzymatic competence to protect cells against iron-mediated peroxide loading.
	GPx [9,19]	Glutathione-dependent peroxide-scavenging enzyme family	↓	↑ Rescued	Proxy tracking functional resilience against ferroptosis and lipid radical survival.
	TAC [7,12]	Holistic representation of cumulative chemical antioxidant defenses in biological fluids	↓	↑ Enhanced	Provides a global macro-level overview of aggregate antioxidant status.
	NO [7,12]	Gaseous signaling radical involved in vasoregulation; scavenged by excess ROS	↓	↑ Stabilized	Evaluates secondary endothelial function and vascular complications of iron overload.
Mitochondrial Bioenergetics	ATP [9,12]	Primary energy currency produced by oxidative phosphorylation	↓	↑ Restored	Used in experimental models to measure metabolic recovery post-treatment.
	ATP/ADP ratio [9]	Reflects immediate cellular bioenergetic charging status and metabolic equilibrium	↓	↑ Improved	Highly sensitive metric of subcellular viability and metabolic restoration.
	Lactate [9]	Anaerobic glycolysis product; elevates when mitochondrial respiration is compromised	↑	↓ Normalized	Clinical and research indicator of localized tissue hypoxia and respiratory chain failure.
	ΔΨm [9,19,48,49]	Electrical gradient across the inner mitochondrial membrane indicating organelle integrity	↓ (depolarized)	↑ Stabilized	Early marker used to track organelle-driven apoptotic and ferroptotic signaling pathways.
	mtDNA copy number [9,12]	Surrogate for organelle genetic pool integrity and mitochondrial population mass	↓	↑ Stabilized	Evaluates levels of oxidative genomic damage and organelle replication efficiency.
	Respiratory complexes I–IV [9,11,49]	Multi-protein enzymatic drivers of the electron transport chain	↓ (impaired)	↑ Improved	Evaluates localized molecular functionality of the metabolic respiratory machinery.
	Citrate synthase [9]	Quantitative enzymatic surrogate representing total mitochondrial mass	↓	↑ Restored	Standard benchmark used to normalize baseline respiratory chain enzyme performance.
Mitochondrial Biogenesis	PGC-1α [9,12]	Master transcriptional coactivator regulating mitochondrial biogenesis pathways	↓	↑ Upregulated	Research marker tracking recovery of cellular capacity to build new mitochondria.
	NRF1 [9]	Transcription factor regulating nuclear genes encoding respiratory subunits	↓	↑ Restored	Tracks upstream genomic signaling responses to mitochondrial network collapse.
	TFAM [9,12]	Drives replication and transcription of the mitochondrial genome	↓	↑ Restored	Used alongside mtDNA assays to assess functional biogenesis signaling.
Ferroptosis Regulation	GPX4 [58,59]	Principal enzyme detoxifying membrane lipid hydroperoxides; central ferroptosis gatekeeper	↓	↑ Preserved	Essential proxy tracking functional resilience against ferroptotic cell death.
	ACSL4 [56,60]	Enzyme shaping cellular lipid composition to promote ferroptosis sensitivity	↑	↓ Reduced	Dictates ferroptosis sensitivity by modulating PUFA-phospholipid metabolism.
	FSP1 [59]	CoQ oxidoreductase acting parallel to GPX4 to inhibit ferroptosis via CoQ reduction	↓ (reduced activity)	↑ Preserved	Parallel ferroptosis defense pathway; targetable vulnerability in iron-mediated injury.

4-HNE stands for 4-hydroxynonenal; 8-OHdG for 8-hydroxy-2′-deoxyguanosine; ACSL4 for acyl-CoA synthetase long-chain family member 4; ADP for adenosine diphosphate; ATP for adenosine triphosphate; CAT for catalase; CoQ for coenzyme Q; FSP1 for ferroptosis suppressor protein 1; GPx for glutathione peroxidase; GPX4 for glutathione peroxidase 4; GSH for reduced glutathione; GSSG for oxidized glutathione; LIC for liver iron concentration; LPI for labile plasma iron; MDA for malondialdehyde; MRI for magnetic resonance imaging; mtDNA for mitochondrial DNA; mtROS for mitochondrial reactive oxygen species; NO for nitric oxide; NRF1 for nuclear respiratory factor 1; NTBI for non-transferrin-bound iron; PGC-1α for peroxisome proliferator-activated receptor gamma coactivator-1 alpha; PUFA for polyunsaturated fatty acid; ROS for reactive oxygen species; SOD for superoxide dismutase; TAC for total antioxidant capacity; TFAM for mitochondrial transcription factor A; and ΔΨm for mitochondrial membrane potential.

**Table 6:** Detailed Database Search Strategies.

Database	Search Strategy
PubMed/MEDLINE	*((“Iron Overload”[Mesh] OR “iron overload” OR “transfusional iron overload” OR “β-thalassemia” OR “myelodysplastic syndromes”) AND (“Mitochondrial Dysfunction” OR “Oxidative Stress”[Mesh] OR “ferroptosis” OR “reactive oxygen species” OR “bioenergetics”) AND (“Chelating Agents”[Mesh] OR “iron chelation” OR deferoxamine OR deferiprone OR deferasirox))*
Embase	*(‘iron overload’/exp OR ‘iron overload’ OR ‘transfusional iron overload’) AND (‘mitochondrial dysfunction’/exp OR ‘oxidative stress’/exp OR ferroptosis OR bioenergetics) AND (‘chelating agent’/exp OR ‘iron chelation’ OR deferoxamine OR deferiprone OR deferasirox)*
Scopus	*TITLE-ABS-KEY (“iron overload” OR “transfusional iron overload” OR β-thalassemia OR “myelodysplastic syndromes”) AND TITLE-ABS-KEY (“mitochondrial dysfunction” OR “oxidative stress” OR ferroptosis OR bioenergetics) AND TITLE-ABS-KEY (“iron chelation” OR deferoxamine OR deferiprone OR deferasirox)*
Web of Science	*TS = (“iron overload” OR “transfusional iron overload” OR β-thalassemia OR “myelodysplastic syndromes”) AND TS = (“mitochondrial dysfunction” OR “oxidative stress” OR ferroptosis OR bioenergetics) AND TS = (“iron chelation” OR deferoxamine OR deferiprone OR deferasirox)*

Emtree, Excerpta Medica Thesaurus; TS, Topic Search; TITLE-ABS-KEY, title, abstract, and keyword fields.

## Data Availability

All data supporting the findings of this systematic review are derived from publicly available, peer-reviewed studies indexed in the searched electronic databases (PubMed/MEDLINE, Embase, the Cochrane Library, and Scopus) and are fully cited within the text. No new datasets or custom software codes were generated or analyzed during the current study. Therefore, additional data sharing is not applicable to this article.

## Supplementary Materials

The following supporting information can be downloaded at: https://www.mdpi.com/article/10.3390/ijms27198657/s1.

## Abbreviations

The following abbreviations are used in this manuscript:

4-HNE	4-Hydroxynonenal
8-OHdG	8-Hydroxy-2′-deoxyguanosine
ACSL4	Acyl-CoA Synthetase Long-Chain Family Member 4
ADP	Adenosine Diphosphate
AI	Artificial Intelligence
ATP	Adenosine Triphosphate
BMP	Bone Morphogenetic Protein
CAT	Catalase
CoQ	Coenzyme Q
DFO	Deferoxamine
DFP	Deferiprone
DFX	Deferasirox
DHODH	Dihydroorotate Dehydrogenase
ELISA	Enzyme-Linked Immunosorbent Assay
EPR	Electron Paramagnetic Resonance
ERFE	Erythroferrone
Fe–S	Iron–Sulfur
FSP1	Ferroptosis Suppressor Protein 1
GPX4	Glutathione Peroxidase 4
GRADE	Grading of Recommendations Assessment, Development and Evaluation
GSH	Reduced Glutathione
GSSG	Oxidized Glutathione
HPLC	High-Performance Liquid Chromatography
IRE	Iron-Responsive Element
IRP	Iron Regulatory Protein
JC-1	5,5′,6,6′-Tetrachloro-1,1′,3,3′-tetraethylbenzimidazolylcarbocyanine iodide
LIC	Liver Iron Concentration
LIP	Labile Iron Pool
LPI	Labile Plasma Iron
MDA	Malondialdehyde
MDS	Myelodysplastic Syndromes
MeSH	Medical Subject Headings
MitoSOX	Mitochondrial Superoxide Indicator
MRI	Magnetic Resonance Imaging
mtDNA	Mitochondrial DNA
mPTP	Mitochondrial Permeability Transition Pore
mtROS	Mitochondrial Reactive Oxygen Species
NCOA4	Nuclear Receptor Coactivator 4
NO	Nitric Oxide
NOS	Newcastle–Ottawa Scale
NRF1	Nuclear Respiratory Factor 1
NTBI	Non-Transferrin-Bound Iron
PGC-1α	Peroxisome Proliferator-Activated Receptor Gamma Coactivator-1 Alpha
PRISMA	Preferred Reporting Items for Systematic Reviews and Meta-Analyses
PROSPERO	International Prospective Register of Systematic Reviews
PUFA	Polyunsaturated Fatty Acid
RCT	Randomized Controlled Trial
RoB 2	Cochrane Risk of Bias 2
ROS	Reactive Oxygen Species
RT-PCR	Reverse Transcription Polymerase Chain Reaction
SOD	Superoxide Dismutase
SYRCLE	Systematic Review Centre for Laboratory Animal Experimentation
TAC	Total Antioxidant Capacity
TBARS	Thiobarbituric Acid Reactive Substances
TFAM	Mitochondrial Transcription Factor A
TFR1	Transferrin Receptor 1
TMRE	Tetramethylrhodamine Ethyl Ester
ΔΨm	Mitochondrial Membrane Potential
