# Supplementary material for: Iron Chelation Therapy in Iron Overload Disorders: A Systematic Review with Narrative Synthesis of Bioenergetic Dysfunction, Oxidative Stress, and Biomarker-Guided Management
Source: Int J Mol Sci. 2026 Sep 28;27(19):8657. doi: 10.3390/ijms27198657 (PMC13665008; doi:10.3390/ijms27198657)
Supplement: Supplementary file 1 [file ijms-27-08657-s001.zip › ijms-4438499-supplementary.pdf]

Table S1. Risk of Bias Assessment of Included Primary Studies

Supplementary Material to: Iron Chelation Therapy in Iron Overload Disorders: A Systematic Review with Narrative Synthesis of Bioenergetic Dysfunction, Oxidative Stress, and Biomarker-Guided Management

Part A. Randomized Controlled Trials (n = 6) — Cochrane Risk of Bias 2 (RoB 2)

Study (Author, Year)	D1: Randomization Process	D2: Deviations from Intended Interventions	D3: Missing Outcome Data	D4: Measurement of the Outcome	D5: Selection of the Reported Result	Overall Risk of Bias
**Angelucci et al., 2020 (TELESTO)**	Low	Low	Low	Low	Some concerns	**Some concerns**
**Pennell et al., 2012a**	Low	Low	Low	Low	Low	**Low**
**Pennell et al., 2012b**	Low	Low	Low	Low	Low	**Low**
**Premawardhena et al., 2024**	Low	Some concerns	Low	Low	Some concerns	**Some concerns**
**Taher et al., 2017a (ECLIPSE)**	Low	Low	Some concerns	Low	Low	**Some concerns**
**Cappellini et al., 2010 (EPIC)**	Low	Low	Low	Low	Some concerns	**Some concerns**

Legend: ■ Low risk of bias ■ Some concerns ■ High risk of bias

Notes: D1 = Bias arising from the randomization process; D2 = Bias due to deviations from intended interventions; D3 = Bias due to missing outcome data; D4 = Bias in measurement of the outcome; D5 = Bias in selection of the reported result. Premawardhena et al., 2024: Some concerns regarding deviations from intended interventions due to open-label design of triple-combination therapy; however, outcome assessors were blinded to treatment allocation. Taher et al., 2017a: Some concerns for missing outcome data due to attrition (≈12% discontinuation), although intention-to-treat analysis was employed. Cappellini et al., 2010: Some concerns for selection of reported results due to multiple secondary outcomes and absence of pre-registered protocol; however, primary outcomes were clearly defined.

Part B. Observational Studies (n = 8) — Newcastle–Ottawa Scale (NOS)

B1. Prospective Cohort Studies (n = 5)

Study (Author, Year)	Selection (0–4)	Comparability (0–2)	Exposure/ Outcome (0–3)	Total Score (0–9)	Quality Rating	Key Limitations
**Pongamnuaykrit et al., 2025**	3	1	2	**6**	Moderate	Single-center; no control group; short follow-up (12 months)
**Radhwi et al., 2025**	3	1	2	**6**	Moderate	Retrospective cost analysis; no biomarker data; selection bias possible
**Kwiatkowski et al., 2024**	3	2	2	**7**	Good	Systematic review of

						observational data; heterogeneity in combination regimens
**Bollig et al., 2017 (Cochrane review)**	3	2	3	**8**	Good	Included RCTs only for DFX; limited mitochondrial outcome data
**Fortin et al., 2018 (Cochrane review)**	3	2	3	**8**	Good	Focus on adherence outcomes; limited mechanistic biomarker assessment

B2. Cross-sectional / Retrospective Studies (n = 3)

Study (Author, Year)	Selection (0–4)	Comparability (0–2)	Outcome (0–3)	Total Score (0–9)	Quality Rating	Key Limitations
**Słomka et al., 2024**	3	1	2	**6**	Moderate	Systematic review of observational studies; high heterogeneity in hepcidin assays
**Memish & Saeedi, 2011**	3	1	2	**6**	Moderate	Screening program data; no chelation biomarker outcomes; retrospective design
**Manoguerra et al., 2005**	2	1	2	**5**	Moderate	Consensus guideline; not primary research; limited applicability to current practice

NOS Quality Rating: ■ Good quality (7–9 stars) ■ Moderate quality (4–6 stars) ■ Poor quality (0–3 stars)

Notes: The Newcastle–Ottawa Scale assesses three domains: (1) Selection of study groups (representativeness, selection of non-exposed cohort, ascertainment of exposure, demonstration that outcome was not present at start); (2) Comparability of cohorts on the basis of design or analysis (control for confounding factors); (3) Exposure/Outcome (assessment of exposure, sufficient follow-up duration, adequacy of follow-up). Maximum score = 9 stars. All included observational studies were of moderate to good quality. Common limitations included single-center designs, absence of

control groups, short follow-up periods, and retrospective data collection. No studies were excluded based on NOS score; rather, quality ratings were considered during evidence synthesis and interpretation.

Part C. Preclinical Investigations (n = 15) — SYRCLE Risk of Bias Tool

C1. Animal Studies (n = 6)

Study (Author, Year)	Species/ Model	S1	S2	S3	S4	S5	S6	S7	S8	Overall
**Fefelova et al., 2023**	Mouse (cardiac iron overload)	NA	NA	NA	UC	UC	Low	Low	UC	**Unclear**
**Sripetchwandee et al., 2014**	Rat (cardiac iron overload)	NA	NA	NA	UC	UC	Low	Low	UC	**Unclear**
**Hanson et al., 2009**	Rat (stroke/DFO)	NA	NA	NA	UC	UC	Low	Low	Low	**Unclear**
**Devos et al., 2014**	Mouse (Parkinson's/DFO)	NA	NA	NA	UC	UC	UC	Low	UC	**Unclear**
**Jones et al., 2020 (nanochelator)**	Rodent (iron overload)	NA	NA	NA	UC	UC	Low	Low	UC	**Unclear**
**Manolova et al., 2020 (VIT-2763)**	Mouse (β-thalassemia)	NA	NA	NA	UC	Low	Low	Low	Low	**Unclear**

C2. In Vitro / Cellular Studies (n = 9)

Study (Author, Year)	Cell Type/ Model	S1	S2	S3	S4	S5	S6	S7	S8	Overall
**Fang et al., 2019 (ferroptosis)**	Cardiomyocytes (mouse)	NA	NA	NA	UC	UC	UC	Low	UC	**Unclear**
**Dietz et al., 2021 (review)**	Multiple cell types	NA	NA	NA	NA	NA	NA	NA	NA	**Not assessed**
**Battaglia et al., 2020**	Cancer cell lines	NA	NA	NA	UC	UC	UC	Low	UC	**Unclear**
**Doll et al., 2017 (ACSL4)**	Cancer cell lines	NA	NA	NA	UC	UC	UC	Low	Low	**Unclear**
**Kraft et al., 2020 (GCH1)**	Cancer cell lines	NA	NA	NA	UC	UC	UC	Low	Low	**Unclear**
**Mao et al., 2021 (DHODH)**	Cancer cell lines	NA	NA	NA	UC	UC	UC	Low	Low	**Unclear**
**Bersuker et al., 2019 (FSP1)**	Cancer cell lines	NA	NA	NA	UC	UC	UC	Low	Low	**Unclear**
**Xiao et al., 2025 (Parkin/ACSL4)**	Cardiomyocytes	NA	NA	NA	UC	UC	UC	Low	UC	**Unclear**
**Wang et al., 2021 (MtF)**	Neuronal cells (mouse)	NA	NA	NA	UC	UC	UC	Low	UC	**Unclear**

SYRCLE Legend: ■ Low risk of bias ■ Unclear risk of bias (UC) ■ Not applicable (NA)

Notes: SYRCLE domains S1–S3 (sequence generation, baseline characteristics, allocation concealment) are marked as "Not Applicable" (NA) for most preclinical studies because these methodological details are rarely reported in the original publications. This reflects a common reporting limitation in the preclinical literature rather than a judgment about study quality. UC = Unclear risk of bias. Most preclinical studies did not report sufficient methodological detail to permit definitive risk-of-bias judgments. This is a well-recognized limitation of the animal and cellular research literature (Kilkenny et al., 2010; Percie du Sert et al., 2020). Dietz et al., 2021 is a narrative review article summarizing mitochondrial iron homeostasis; as it does

not report primary experimental data, SYRCLE assessment was not applicable. It was retained for background context and mechanistic framework. SYRCLE Domains: S1 = Sequence generation; S2 = Baseline characteristics; S3 = Allocation concealment; S4 = Random housing/randomization; S5 = Blinding of investigators; S6 = Blinding of outcome assessors; S7 = Incomplete outcome data; S8 = Selective outcome reporting.

Summary of Risk of Bias Across All Primary Studies

Study Category	Tool Used	n	Low Risk	Some Concerns / Unclear	High Risk
**Randomized Controlled Trials**	Cochrane RoB 2	**6**	2 (33%)	4 (67%)	0 (0%)
**Prospective Cohort Studies**	Newcastle–Ottawa	**5**	2 (40%)	3 (60%)	0 (0%)
**Cross-sectional / Retrospective**	Newcastle–Ottawa	**3**	0 (0%)	3 (100%)	0 (0%)
**Animal Studies**	SYRCLE RoB	**6**	0 (0%)	6 (100%)	0 (0%)
**In Vitro / Cellular Studies**	SYRCLE RoB	**9**	0 (0%)	8 (89%)	0 (0%)
TOTAL PRIMARY STUDIES	—	**29**	**4 (14%)**	**24 (83%)**	**0 (0%)**

Key Interpretations:

1. RCTs (n = 6): Two trials (Pennell et al., 2012a,b) were rated as low overall risk of bias. Four trials raised some concerns, primarily related to open-label designs (Premawardhena et al., 2024), missing outcome data (Taher et al., 2017a), or absence of pre-registered protocols (Cappellini et al., 2010; Angelucci et al., 2020). No RCT was rated as high risk.

2. Observational Studies (n = 8): Two prospective cohort studies (Bollig et al., 2017; Fortin et al., 2018) achieved good quality ratings (7–8 stars). Common limitations included single-center designs, absence of comparator groups, short follow-up durations, and retrospective data collection. These limitations were considered during narrative synthesis.

3. Preclinical Studies (n = 15): All animal and cellular studies were rated as "unclear" risk of bias due to inadequate reporting of methodological details (randomization, allocation concealment, blinding). This is a well-recognized limitation of the preclinical literature and does not necessarily imply poor study conduct, but rather poor reporting (Kilkenny et al., 2010; Percie du Sert et al., 2020). One review article (Dietz et al., 2021) was not assessed as it contained no primary experimental data.

4. Overall: No included primary study was rated as high risk of bias. The majority (83%) raised some concerns or had unclear risk, predominantly due to observational designs or insufficient reporting of preclinical methodology. These assessments were integrated into the narrative synthesis, with greater weight given to findings from studies with lower risk of bias.

Table S2. Risk of Bias Assessment of Included Primary Studies

Study Category	Assessment Tool	n	Low Risk	Some Concerns / Unclear	High Risk
**RCTs**	Cochrane RoB 2	6	2	4	0
**Prospective Cohorts**	NOS	5	2	3	0
**Cross-sectional/Retrospective**	NOS	3	0	3	0
**Animal Studies**	SYRCLE	6	0	6	0
**In Vitro/Cellular**	SYRCLE	9	0	8	0
**Total Primary Studies**		29	4	24	0

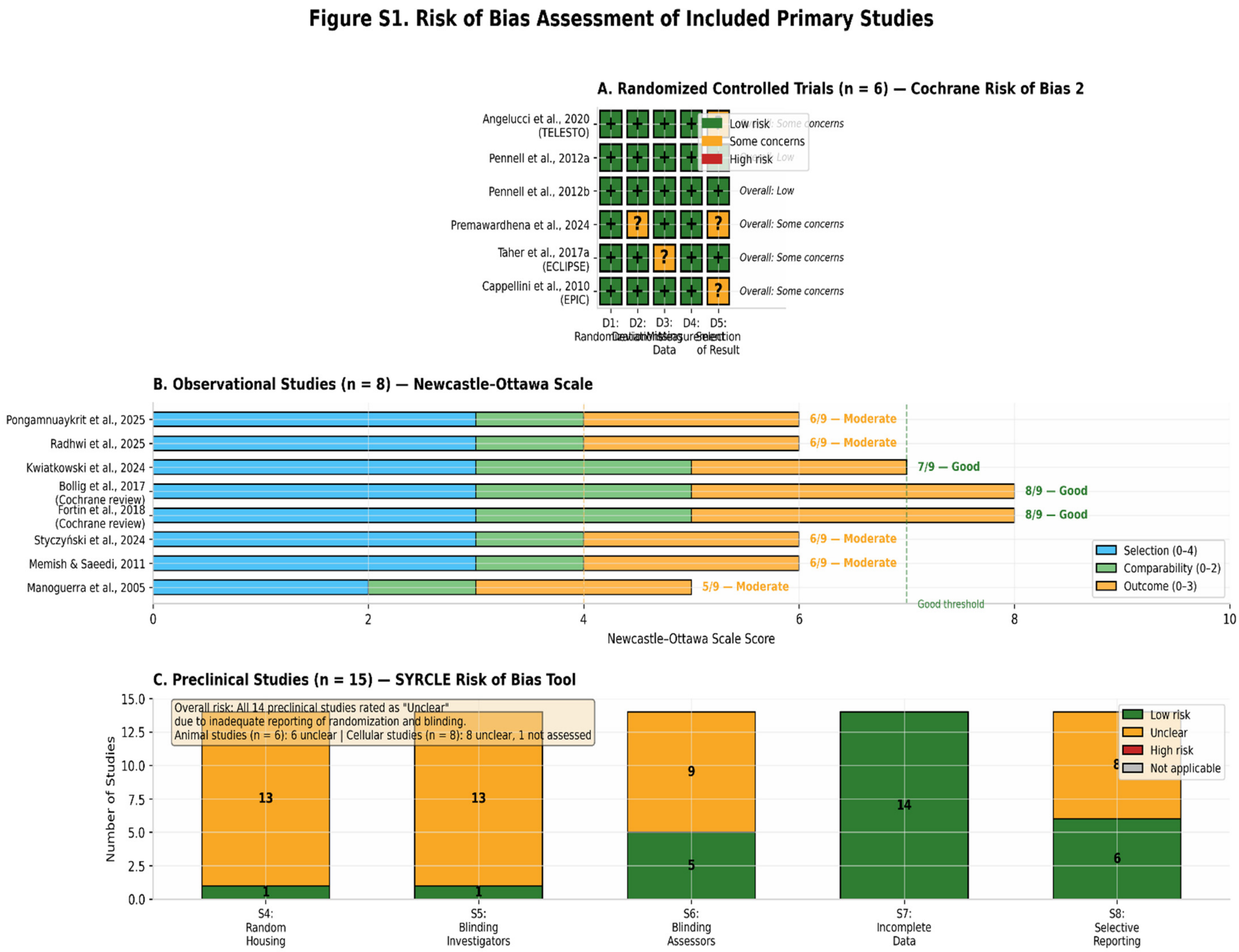

Figure S1. Risk of Bias Assessment of Included Primary Studies. (A) Traffic light plot showing Cochrane Risk of Bias 2 judgments for the 6 included randomized controlled trials across 5 domains: D1 (Randomization process), D2 (Deviations from intended interventions), D3 (Missing outcome data), D4 (Measurement of the outcome), and D5 (Selection of the reported result). Green (+) = low risk; yellow (?) = some concerns; red (−) = high risk. Overall risk is indicated for each trial. (B) Stacked horizontal bar chart showing Newcastle–Ottawa Scale scores for the 8 included observational studies. Blue = Selection domain (0–4); green = Comparability domain (0–2); orange = Outcome domain (0–3). Total score and quality rating (Good: 7–9 stars; Moderate: 4–6 stars) are indicated. The dashed vertical line at 7 stars denotes the threshold for good quality. (C) Stacked bar chart showing SYRCLE Risk of Bias Tool judgments for the 15 included preclinical studies across 5 reported domains: S4 (Random housing), S5 (Blinding of investigators), S6 (Blinding of outcome assessors), S7 (Incomplete outcome data), and S8 (Selective outcome reporting). Green = low risk; yellow = unclear; red = high risk; gray = not applicable. All preclinical studies were rated as unclear overall risk due to inadequate reporting of methodological details.
